# Transcriptional Mechanisms of Proneural Factors and REST in Regulating Neuronal Reprogramming of Astrocytes

**DOI:** 10.1016/j.stem.2015.05.014

**Published:** 2015-07-02

**Authors:** Giacomo Masserdotti, Sébastien Gillotin, Bernd Sutor, Daniela Drechsel, Martin Irmler, Helle F. Jørgensen, Steffen Sass, Fabian J. Theis, Johannes Beckers, Benedikt Berninger, François Guillemot, Magdalena Götz

**Affiliations:** 1 Physiological Genomics, Biomedical Center, University of Munich, 80336 Munich, Germany; 2 Institute for Stem Cell Research, Helmholtz Centre Munich, 85764 Neuherberg, Germany; 3 The Francis Crick Institute, Mill Hill Laboratory, The Ridgeway, London, NW7 1AA, UK; 4 Hutchison/MRC Research Center, University of Cambridge, Cambridge Biomedical Campus, Cambridge, CB2 0XZ, UK; 5 Institute of Experimental Genetics, Helmholtz Centre Munich, 85764 Neuherberg, Germany; 6 Department of Medicine, University of Cambridge, Cambridge, CB2 0QQ, UK; 7 Institute of Computational Biology, Helmholtz Centre Munich, 85764 Neuherberg, Germany; 8 Department of Mathematics, Technical University Munich, 85748 Garching, Germany; 9 Institute of Physiological Chemistry, University Medical Center of the Johannes Gutenberg University, D-55128 Mainz, Germany; 10 Focus Program Translational Neuroscience, Johannes Gutenberg University, D-55128 Mainz, Germany; 11 Center of Life and Food Sciences Weihenstephan, Technical University, 85354 Freising, Germany; 12 Munich Cluster for Systems Neurology “SyNergy,” Ludwig Maximilian University of Munich, 80539 Munich, Germany

## Abstract

Direct lineage reprogramming induces dramatic shifts in cellular identity, employing poorly understood mechanisms. Recently, we demonstrated that expression of Neurog2 or Ascl1 in postnatal mouse astrocytes generates glutamatergic or GABAergic neurons. Here, we take advantage of this model to study dynamics of neuronal cell fate acquisition at the transcriptional level. We found that Neurog2 and Ascl1 rapidly elicited distinct neurogenic programs with only a small subset of shared target genes. Within this subset, only NeuroD4 could by itself induce neuronal reprogramming in both mouse and human astrocytes, while co-expression with Insm1 was required for glutamatergic maturation. Cultured astrocytes gradually became refractory to reprogramming, in part by the repressor REST preventing Neurog2 from binding to the NeuroD4 promoter. Notably, in astrocytes refractory to Neurog2 activation, the underlying neurogenic program remained amenable to reprogramming by exogenous NeuroD4. Our findings support a model of temporal hierarchy for cell fate change during neuronal reprogramming.

## Highlights

• Neurog2 and Ascl1 regulate largely non-overlapping neurogenic targets
• A subset of genes is required and sufficient to convert astrocytes and fibroblasts
• Neurog2 and REST compete for binding to the *NeuroD4* promoter
• REST deletion enhances Neurog2-mediated reprogramming in vitro

## Graphical Abstract

**Figure:**
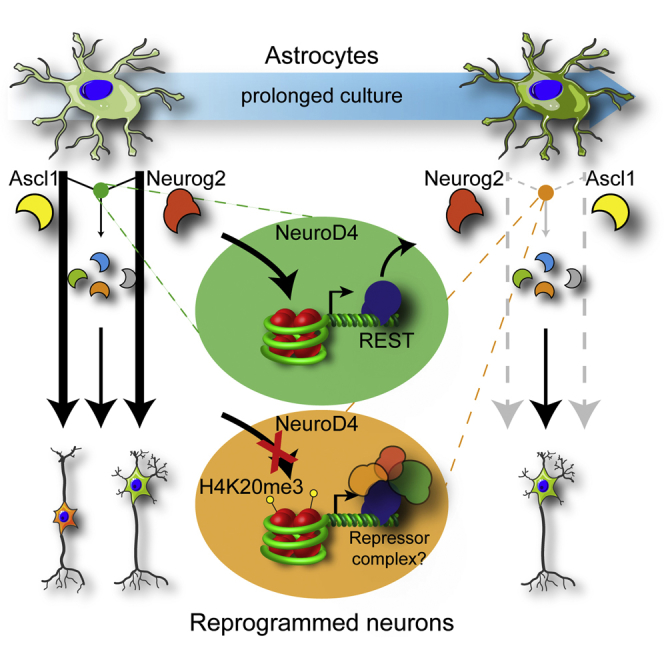

## Teaser

Masserdotti et al. analyzed early transcriptional changes mediated by Neurog2 and Ascl1 during direct reprogramming of murine postnatal astrocytes into distinct neuronal subtypes in vitro. This led to the identification of shared downstream targets, including NeuroD4, capable of neuronal reprogramming of fibroblasts and human astrocytes, as well as mechanistic insight into how the repressor REST functions as a barrier in direct neuronal reprogramming.

## Introduction

During development, neuronal subtypes are generated typically in distinct regions with patterning cues initiating regional programs of neurogenesis (Martynoga et al., 2012). In the telencephalon, for example, stem and progenitor cells in the ventral region are instructed to express the transcription factors Ascl1, Gsx1/2, and Dlx1/2, which then regulate the specification of GABAergic projection and interneurons (for review see Imayoshi and Kageyama, 2014); in the dorsal telencephalon, progenitors express different transcription factors, such as Emx1/2, Pax6, and Neurog1/2, which regulate the specification of glutamatergic projection neurons (Schuurmans and Guillemot, 2002). Analysis of the transcriptional programs in mouse mutants revealed rather distinct transcriptional targets regulated by these transcription factors in the dorsal and ventral telencephalon (Gohlke et al., 2008). Whether this limited overlap is due to early divergence of these regions initiated by patterning signals, resulting in distinct transcriptional contexts, remains an open question. Neurons may be specified in a hierarchical manner, with the induction of common neuronal traits first, followed later by neuronal subtype features via a final set of transcription factors, such as terminal selector genes (Hobert, 2011). Conversely, distinct transcriptional regulators may specify different neuronal subtypes already at the onset of neuronal commitment, with relatively little overlap between transcriptional programs.

Direct reprogramming is especially well suited to examine the programs elicited by distinct transcription factors within the same cellular and epigenetic context. When expressed in astrocytes obtained from postnatal murine cerebral cortex gray matter, Ascl1 instructs GABAergic neurons, while Neurog2 elicits glutamatergic neurons (Berninger et al., 2007; Heinrich et al., 2010), thus making possible the identification of target genes involved in neuronal subtype specification within the same transcriptional background. In different cell types, such as fibroblasts, Ascl1 induces a glutamatergic neuronal fate in combination with Myt1L and Brn2 in fibroblasts (Vierbuchen et al., 2010), while Neurog2 forces motor neuron generation in combination with forskolin and dorsomorphin (Liu et al., 2013). Thus, the cell of origin, with its specific epigenetic landscape, can play a role in defining the spectrum of reprogramming possibilities.

To date, the transcriptional programs elicited by direct lineage conversion toward neuronal fates are still largely elusive. Emerging evidences suggest an important role for epigenetic mechanisms as a hurdle to reprogramming (Wapinski et al., 2013; Xue et al., 2013). Large repressive protein complexes have been implicated in cell fate specification and differentiation: for instance, the REST/CoREST complex, known for its role in maintaining neural stem cells (Laugesen and Helin, 2014) and neuronal differentiation (Lu et al., 2014) has been shown to be the target of miRNA-mediated reprogramming of fibroblast into neurons (Xue et al., 2013). However, is it known neither when and how REST contributes to repress direct reprogramming, nor the mechanisms relevant in establishing reprogramming borders during cell differentiation.

To tackle these important questions, we examined the temporal regulation of genes at early stages of in vitro direct reprogramming of young postnatal astrocytes into neurons using tamoxifen-inducible forms of Ascl1 and Neurog2, which allowed the unraveling of the dynamics of transcriptional regulation as well as an understanding of the mechanisms involved in the failure to activate key targets in unresponsive astrocytes.

## Results

### Activation of Neurog2ERT2 and Ascl1ERT2 Instructs Neurons from Glia

**Figure 1:**
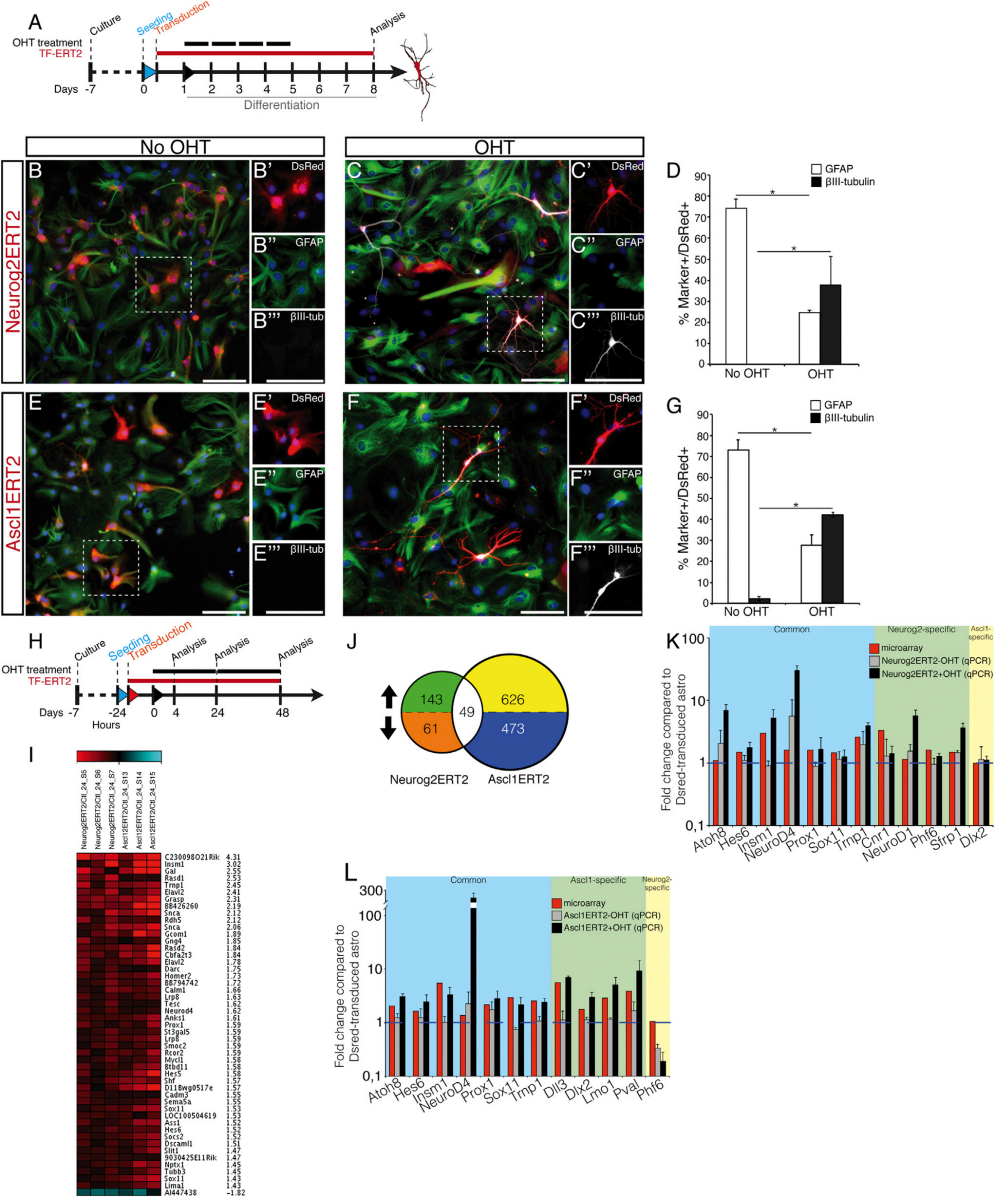
Temporal Analysis of Genome-wide Transcription Changes in Astrocyte Reprogramming (A) Schematic representation of the experimental procedure inducing the activation of Neurog2ERT2-IRES-DsRed or Ascl1ERT2-IRES-DsRed by tamoxifen (OHT indicated by uppermost black bars) for reprogramming astrocytes into neurons. (B, C, E, and F) Micrographs of astrocytes infected with the constructs indicated in red on the left side and immunostained for the astrocytic marker GFAP (green) and the neuronal marker βIII-tubulin (white). Scale bars, 100 μm. (D and G) Quantification of non-reprogrammed cells (GFAP) or reprogrammed cells (βIII-tubulin) without or with OHT 8 days post-induction (DPI). Mean ± SEM; n = 4 independent experiments; statistical test: two-tailed Mann-Whitney test (^∗^p < 0.05). (H) Schematic representation of the experimental procedure for genome-wide mRNA analysis. (I) Heatmap of genes regulated by both Neurog2ERT2 and Ascl1ERT2 within 24 hr after induction by OHT. (J) Venn diagram of genes regulated by Neurog2ERT2 or Ascl1ERT2 24 hr after OHT. (K and L) Real-time qPCR) analysis on selected candidates upon Neurog2ERT2 (K) or Ascl1ERT2 (L) induction by OHT for 24 hr. Mean ± SEM; n = 3 independent experiments. See also Figure S1, Table S1, Table S2, Table S3, Table S4, and Table S5.

In order to investigate the early events of direct reprogramming, the cDNA of *Neurog2* and *Ascl1* was fused to the modified estrogen receptor ligand binding domain ERT2 (Raposo et al., 2015) and sub-cloned into a retroviral construct, together with the red fluorescent protein (DsRed-Expressed2, hereafter indicated as DsRed) (Berninger et al., 2007; Heinrich et al., 2010; Heins et al., 2002). Proliferating astrocytes were obtained from postnatal day (P)6–7 mouse cerebral cortex Gray Matter (GM), avoiding the White Matter (WM) and ventricular regions comprising endogenous neural stem cells (Imura et al., 2006). The purity of these cultures was previously assessed with various astrocytic markers and genetic fate mapping (Berninger et al., 2007; Heinrich et al., 2010; Heins et al., 2002) (see also Figures S1I and S1J). Moreover, cells infected with control retroviral vectors expressing GFP or DsRed showed a low proportion of Lewis X+ progenitors (3.9% ± 1.6% at day 2, Figures S1A–S1H) and did not generate any βIII-tubulin+ neurons (0%, 250 cells counted/experiment, n = 8). Likewise, Neurog2ERT2-transduced or Ascl1ERT2-transduced cells remained GFAP+ and generated virtually no neurons after 1 week without 4-hydroxy-tamoxifen (OHT) addition (Figures 1B and 1E; quantification in Figures 1D and 1G, 0% with Neurog2ERT2 and 0.8% with Ascl1ERT2; Figures 1D and 1G). Thus, these cultures contain largely non-neurogenic proliferating astrocytes.

Treatment with OHT for 4 consecutive days elicited the highest efficiency of neuronal conversion, as assessed by morphology and βIII-tubulin immunostaining (Figures 1C and 1F; quantification in Figures 1D and 1G; for shorter periods see Figures S1K–S1M). Importantly, this OHT treatment of Neurog2ERT2- and Ascl1ERT2-transduced astroglia triggered similar reprogramming efficiency (40% of DsRed+ cells), thus providing a suitable system for the investigation of the transcriptional changes during reprogramming triggered by the two factors.

### Ascl1 and Neurog2 Induce Rapid but Distinct Transcriptional Programs in Astrocytes In Vitro

First, we analyzed the transcriptome of Neurog2ERT2- and Ascl1ERT2-transduced astroglial cultures after OHT-treatment for 4, 24, and 48 hr (Figure 1H; Figures S1N and S1O′ for transduction efficiency). Activation of Neurog2ERT2 for 4 hr changed the expression of 199 probesets (fold change > 1.2, rawp (p value of the t-test statistics) < 0.01, Table S1), suggesting that transcriptional changes take place rapidly. This set of regulated genes was significantly enriched for the gene ontology (GO) terms (Huang et al., 2009) (as codified according to DAVID; http://david.abcc.ncifcrf.gov) associated with “regulation of cell proliferation,” “cell adhesion,” and, despite the early stage, “voltage-gated channel activity,” including genes expressed by excitable cells, such as *Scn8a* and *Cacna1d* (Table S2). At 24 hr, the number of Neurog2ERT2-regulated probesets increased further by 27% (253, fold change > 1.2, rawp < 0.01, Figure S1P, Table S1). At 48 hr a different group of probesets was regulated (Figure S1P, Table S1), such that only 6% of the 712 probesets regulated during the period analyzed showed an altered expression at two or more time points (43 probesets, Figure S1P). Thus, large-scale and dynamic changes in gene expression take place throughout the first 48 hr of direct neuronal reprogramming.

We then examined the transcriptome changes upon Ascl1ERT2 activation, which resulted in a higher number of regulated genes, possibly due to Ascl1 acting as a master and pioneering transcription factor (Wapinski et al., 2013). Rapid changes in gene expression were observed already at 4 hr (621 probesets, GO terms in Table S4), increased at 24 hr (1,148 probesets), and decreased at 48 hr (591 probesets; fold change > 1.2, rawp < 0.01, Figure S1P′, Table S3). Overall, 13.5% of all the probesets altered at any time point after Ascl1ERT2 activation were significantly regulated at two time points at least (319 out of 2,360 probesets, Figure S1P′, Table S3). Thus, Ascl1ERT2 also induced fast and dynamic changes in gene expression, suggesting a rapid change in cellular identity.

Of the probesets regulated by Neurog2ERT2 or Ascl1ERT2, only 1.34% was common to both factors at 4 hr after induction (Figure S1Q), 3.5% at 24 hr (Figures 1I and 1J), and 3.1% at 48 hr (Figure S1R). Overall, the probesets regulated by both transcription factors account for only 2.8% of all the probesets regulated at any time by either factor, demonstrating that the small overlap is not due to different kinetics of the Neurog2- and Ascl1-induced programs, but rather to the activation of largely different gene cascades.

GO terms associated with the small subset of targets common at 24 hr (49 probesets) were enriched for the terms “neuronal development” and “neurogenesis” (Figure S1S, Table S5) with 79% of them expressed in neurons and progenitors throughout the brain and 61% with a pan-neuronal expression (such as *Atoh8*, *Hes6*, *Insm1*, *NeuroD4*, *Prox1*, *Sox11*, and *Trnp1*; see Table S6). Selected candidates downstream of Neurog2ERT2 and Ascl1ERT2 were validated by real-time qPCR at 24 hr (Figures 1K and 1L). The expression of *Dlx2*, a known target of Ascl1 (Poitras et al., 2007), was unaffected by Neurog2ERT2 (Figure 1K), and expression of *Phf6*, a Neurog2-regulated gene (Voss et al., 2007), was reduced after Ascl1ERT2 activation (Figure 1L), confirming that the overexpression of these factors in astrocytes did not affect their target specificity.

### Identification of Target Genes Crucial for the Reprogramming of Astroglial Cells

**Figure 2:**
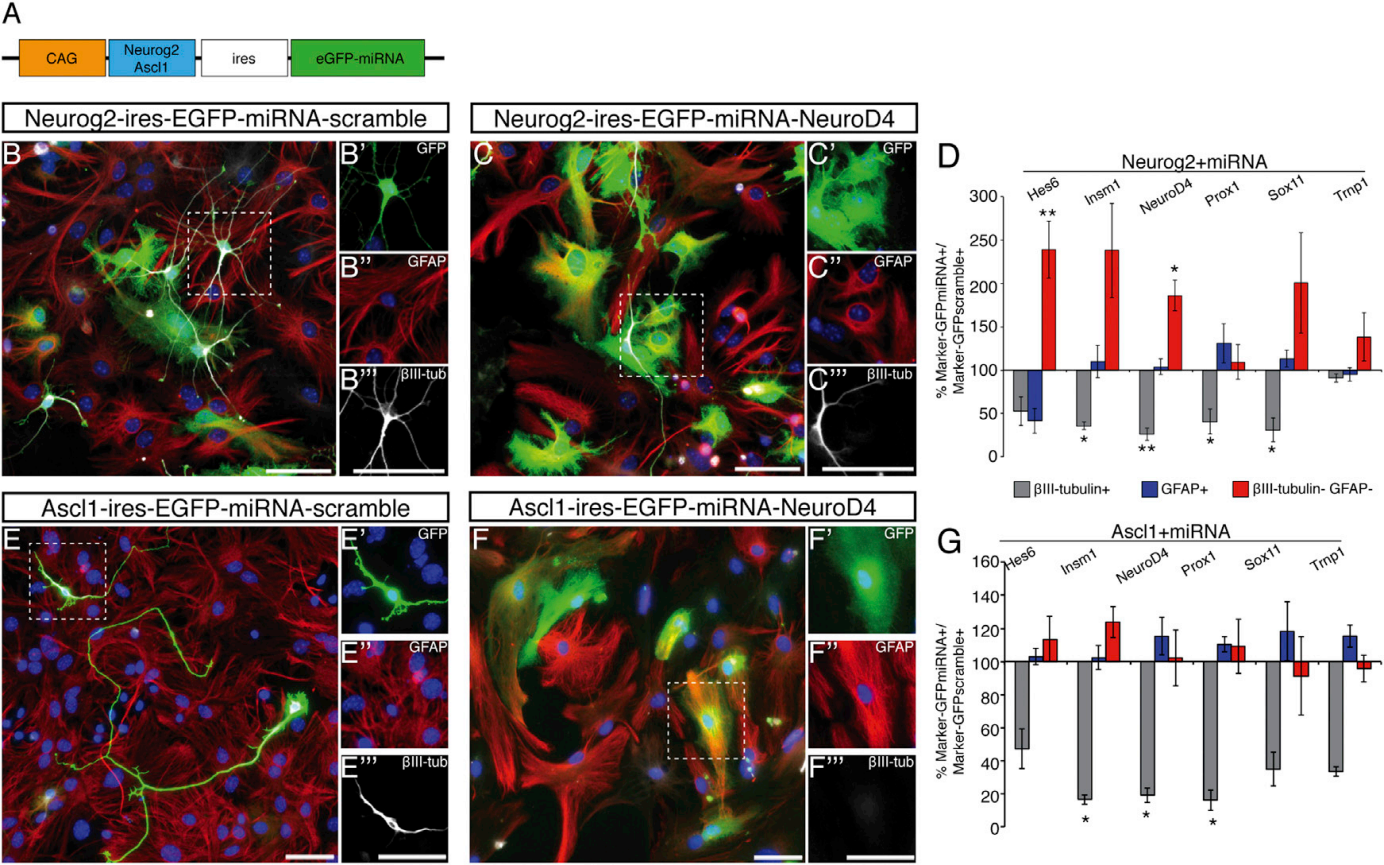
Identification of Essential Downstream Effectors in Astrocyte Reprogramming (A) Schematic representation of retrovirus with expression cassette for miRNAs. (B, C, E, and F) Micrographs of astrocytes, infected with the vectors indicated on top of the panels (green), were immunostained for GFAP (red) and βIII-tubulin (white). Scale bars, 50 μm. (D and G) Quantification of changes in βIII-tubulin+ neurons (gray bars), GFAP+ astrocytes (blue bars) or double-negative cells (red bars) at 8 DPI with the vectors indicated on top of the histograms. Mean ± SEM in (D); n = 4 independent experiments (^∗^p < 0.05; ^∗∗^p < 0.01). Mean ± SEM in (G); n = 3 independent experiments (^∗^p < 0.05). See also Figure S2.

To examine the contribution of the common downstream targets during reprogramming, we designed miRNAs against a subset of these candidates (most efficient in red; Figure S2). While astrocytes transduced with a construct co-expressing Neurog2 and a miRNA-scramble control gave rise to a substantial number of βIII-tubulin+ neurons (Figures 2A and 2B–2B′′′), much fewer neurons were generated upon a specific miRNA’s co-expression (Figures 2C–2C′′′; example with miRNA-NeuroD4). All Neurog2-IRES-miRNA constructs except for Neurog2-IRES-miRNA-Trnp1 reduced the proportion of neurons among infected cells to almost 50% or less compared to Neurog2-miRNA-scamble control (Figure 2D). The percentage of GFAP+ cells did not change significantly between gene-specific and scramble-miRNAs (Figure 2D) with the exceptions of miRNA-Hes6 and miRNA-Prox1. Upon knockdown of *Hes6*, *Insm1*, and *NeuroD4,* the proportion of GFAP-βIII-tubulin double negative cells increased among GFP-labeled cells (Figure 2D), suggesting that some transduced cells might have undergone partial reprogramming.

Consistent with the selected factors acting also downstream of Ascl1, miRNAs against *Insm1*, *NeuroD4*, and *Prox1* reduced the proportion of neurons induced by Ascl1 in astrocytic cultures to 20% or less of the proportion of neurons found in Ascl1-miRNA-scrambled-transduced cultures and to 50% or less for *Hes6*, *Sox11*, and *Trnp1* (Figures 2E–2E′′′ and F–F′′′, quantification in Figure 2G). Little increase in GFAP+ or double negative cells was observed in cultures transduced with Ascl1-specific miRNA viruses (Figures 2E and 2G). Thus, Ascl1 represses the astrocyte fate independently of the selected neurogenic targets, in contrast to Neurog2 (Figure 2D).

Together these data indicate that few commonly regulated neurogenic transcription factors are essential contributors to the reprogramming process.

### Direct Neuronal Reprogramming by Downstream Effectors

**Figure 3:**
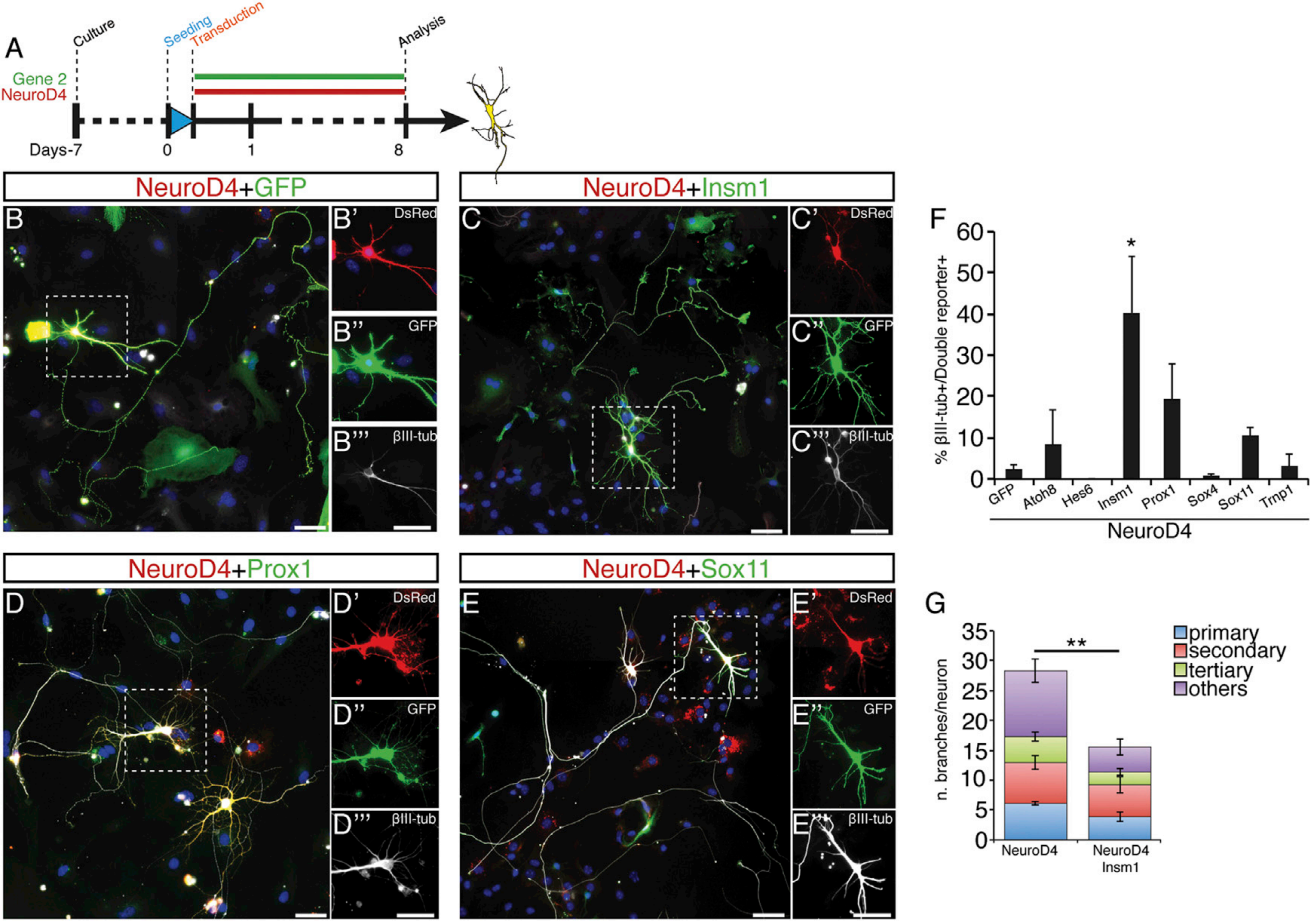
Combinations of Common Downstream Targets Reprogram Astrocytes into Neurons (A) Schematic representation of the experimental procedure. (B–E) Micrographs depicting astrocytes co-infected with the constructs indicated on top of the panels (red and green) immunostained for βIII-tubulin (white) at 8 DPI. Scale bars, 50 μm. (F) Quantification of βIII-tubulin+ cells with neuronal morphology among DsRed+GFP+ double infected cells at 8 DPI. Mean ± SEM; n = 4 independent experiments (^∗^p < 0.05). (G) Quantification of branches per neurons/combination. Mean ± SEM; n = 3 independent experiments (^∗∗^p < 0.01). See also Figures S3 and S4.

To test whether the selected downstream transcription factors could elicit neuronal reprogramming on their own, we combined the expression of three genes at time and found NeuroD4 (ND4) present in all of the pools inducing neurons at 8 days post-transduction (DPT) (Figure S3A). Moreover, ND4 alone was sufficient to induce a small but consistent fraction of βIII-tubulin+ neuronal cells (1%–3%, Figures 3A and 3F), while none of the other factors succeeded in doing so (data not shown). With the combination of two factors, ND4 was most efficient in eliciting neuronal conversion with Insm1 (I), Prox1 (P), or Sox11 (S11) (Figures 3C–3F). Reprogrammed cells showed a distinct neuronal morphology with elaborated dendrites and a long thin process, reminiscent of an axon (Figures 3B–3E). ND4-induced neurons had more branched neurites than NeuroD4-Insm1-induced neurons (ND4+I), suggesting that these might be distinct neuronal subtypes (Figure 3G).

**Figure 4:**
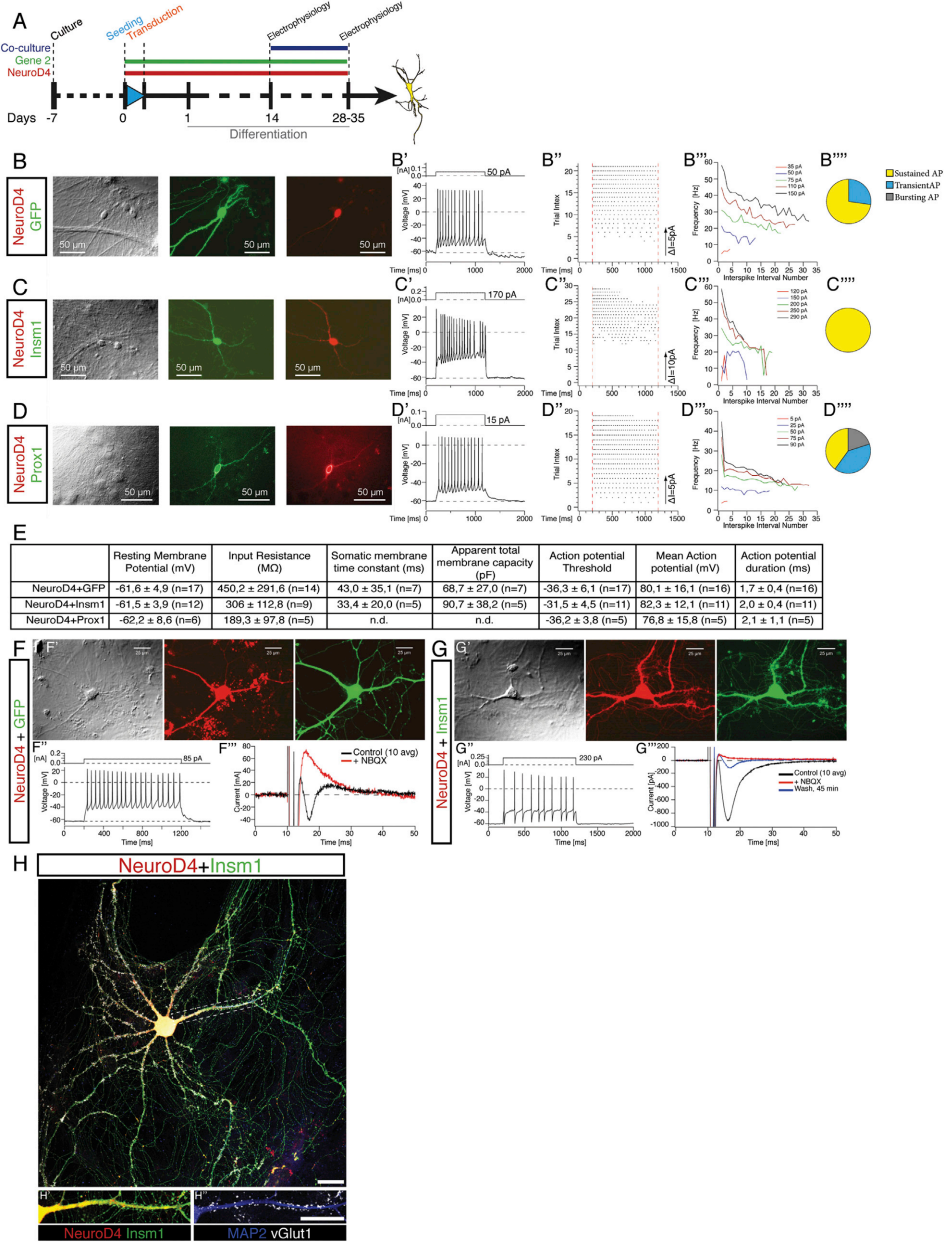
Generation of Synaptically Mature Neurons upon Combined Expression of Downstream Targets (A) Schematic representation of the experimental procedure. (B–D) Electrophysiological characterization of induced neurons upon overexpression of the constructs indicated by live fluorescence during recordings. Examples of sustained trains of APs generated when recording in current-clamp mode are shown (in B′, C′, and D′ top panel: stimulation protocol). 50% repetitive firing NeuroD4/GFP cells present first spike latency lower than 70 ms, with 50% higher than 150 ms; an example of frequency adaptation is shown (B′′ and B′′′). In (C′), an example of a repetitive AP generated in NeuroD4/Insm1 transduced cells is shown (four generated the first spike with a latency lower than 70 ms and the remaining two did so with a latency higher than 150 ms) and characterized by spike accommodation (C′′) and spike adaptation (C′′′). (D′′ and D′′′) show examples of repetitive spike discharge in NeuroD4+Prox1-expressing neurons. (B′′′′–D′′′′) A pie chart shows the fraction of cells firing bursting (gray), transient (blue), or sustained (yellow) APs. (E) Table summarizing the electrophysiological parameters measured (brackets indicate the number of cells analyzed). (F) Example of NeuroD4-induced neurons at 14 DPT (F′). A depolarizing current pulse (1 s, 85 pA) induced a train of APs (F′′). In (F′′′) the autaptic response (black trace, average of 10) could be blocked by NBQX (5 μM, red trace, average of 10). (G and G′) Example of NeuroD4-Insm1-induced neurons at 14 DPI. A depolarizing current pulse (1 s, 230 pA) induced a train of APs (G′′). In (G′′′) the autaptic responses (black trace, average of 10) could be blocked by NBQX (10 μM, 10 min red trace, average of 10) and partially reversed following washout for 45 min (blue trace). (H) Micrograph depicting a neuron induced by co-expression of NeuroD4-containing viral vector (red) and Insm-containing viral vector (green) immunostained at 30 DPI for MAP2 (H′, blue) and vGlut1 (H′′, white). Scale bar, 50 μm. See also Figure S3.

To determine whether reprogrammed neuronal cells acquired a genuine neuronal identity, cells transduced with the most efficient combinations of target genes (ND4+I, ND4+P, and ND4 as control; Figures 4B–4D) were analyzed by patch-clamp recording at 28–35 DPI after a 2-week co-culture with cells derived from cerebral cortex at embryonic day (E)14.5 (Figure 4A). All cells with neuronal morphology recorded upon ND4 expression (17/17, Figure 4B, n = 5 independent experiments) or ND4+I (11/11, Figure 4C, n = 3) generated action potentials (APs) upon receiving an injection of suprathreshold current pulses, whereas only 45.5% (5/11, Figure 4D, n = 2) of ND4+P co-transduced neurons fired an AP. Analysis of neuronal properties, such as resting membrane potential, input resistance, somatic membrane time constant, AP threshold, and mean AP amplitude (summarized in Figure 4E) confirmed the functional neuronal nature of reprogrammed cells. ND4-transduced neurons responded to injection of suprathreshold current pulses (1 s) with repetitive spike discharges (example in Figure 4B′′′) associated with frequency adaptation in 72% of cases (8/11, Figures 4B′′ and 4B′′′), as did ND4+I neurons (6/6; example in Figure 4C′′ and 4C′′′) and ND4+P neurons (5/5, Figures 4D′′ and 4D′′′; for higher variability see pie chart in Figure 4D′′′). Interestingly, this pattern resembles that of regular spiking neurons recorded in acute slices of the cerebral cortex (Zolles et al., 2009).

As proof of principle, ND4-reprogrammed neurons were recorded during pharmacological treatments: for instance, addition of TTX (0.5 μM, n = 3) to the bathing solution reversibly blocked the spike induction in ND4 cells (Figure S3B), suggesting that the APs were generated by the activation of voltage-dependent Na+ channels. Moreover, all cells received strong spontaneous synaptic input (Figure S3C, left graphs), either GABAergic, as hyperpolarizing potentials or outward currents recorded under voltage-clamp conditions at −60mV could be as reversibly inhibited by the GABA_a_ receptor antagonist bicuculline (10 μM) (Figure S3C, middle trace), or glutamatergic, as revealed by reversible blockage by the AMPA-receptor antagonist NBQX (10 μM) (Figure S3C, right trace).

As the above data demonstrate that reprogrammed neurons receive functional synapses, we next examined whether they were also competent to form synapses by recording ND4 or ND4+I reprogrammed neurons in the absence of (E)14.5 primary neuronal co-cultures. Already at 8 DPT both ND4 and ND4+I neurons were able to form functional synapses as indicated by the existence of autaptic responses. In ND4-induced neurons, step-depolarizations during voltage-clamp recordings to membrane potentials of −10mV to 0mV elicited autaptic currents (2/11 neurons recorded), which were blocked by NBQX (5–10 μM), thus indicating that these autaptic responses were mediated by synaptically released glutamate via the AMPA-receptor (Figure 4F). Of 12 ND4+I-induced neurons tested, 8 showed autaptic responses that were in all tested cases (5/5) glutamatergic, as they were suppressed by NBQX (5–10 μM; reversible after a prolonged washout period, n = 1, Figure 4G) but not by the GABA_A_ receptor antagonist bicuculline (n = 4). In agreement with electrophysiological data, ND4+I neurons were immunopositive for the synaptic vesicular glutamate transporter vGluT1 (Figure 4H). Thus, the common factors ND4+I induce a glutamatergic neuronal phenotype from cerebral cortex astrocytes.

### NeuroD4 and Insm1 Reprogram Murine Fibroblasts and Human Astrocytes

To determine whether the identified combinations of downstream proneural targets also have a reprogramming activity in other cell types, we expressed them in mouse embryonic fibroblasts (MEFs) (Vierbuchen et al., 2010) and human astrocyte cultures (same cells as in Guo et al., 2014). In MEFs, only ND4+I generated βIII-tubulin+ cells at 14 DPT (Figures S4A–S4D, quantification in Figure S4E), while ND4 alone (Figure S4C) or in combination with other targets failed to do so (data not shown). In human astrocyte cultures (Figures S4F and S4G′), βIII-tubulin+ cells appeared in samples transduced with ND4 alone or in combination with the selected genes already at 8 DPT (Figures S4I–S4L, quantification in M), but not in control cells (Figure S4H). Thus, these downstream transcription factors are also sufficient to reprogram cells from other species or germ layers.

### Astroglia Reprogramming Is Impaired when Neurog2ERT2 Activation Is Delayed

**Figure 5:**
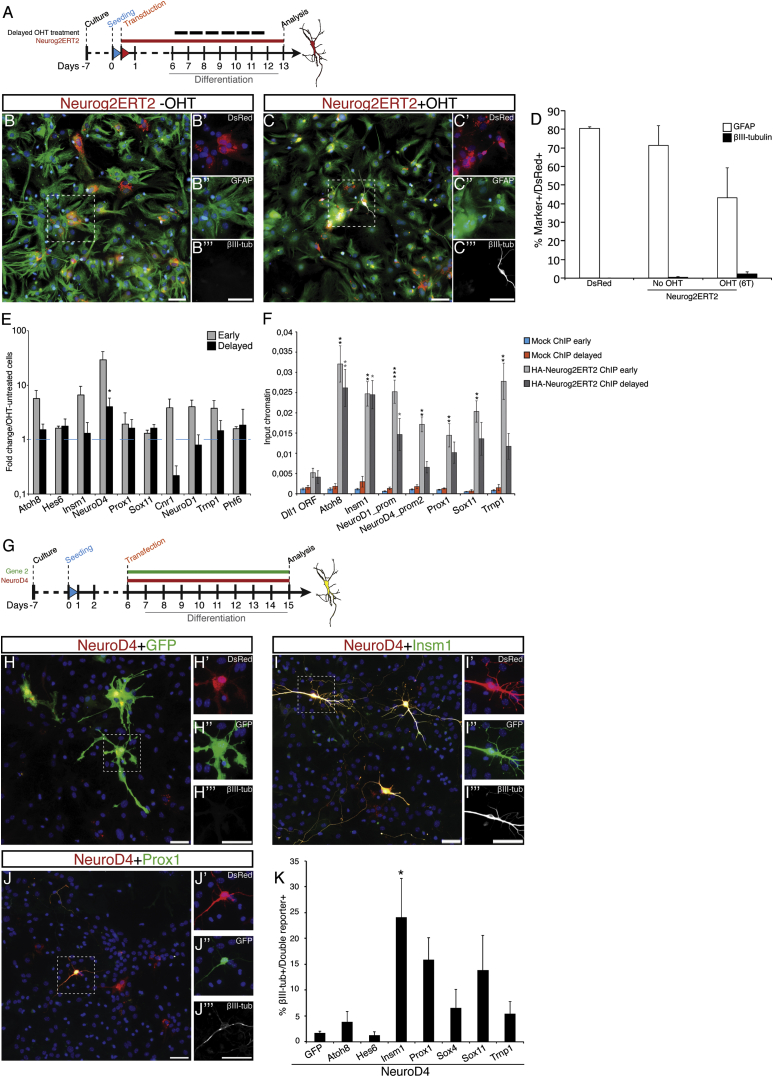
Delayed Induction of Neurog2ERT2 Reveals a Block in Astrocyte Reprogramming (A) Scheme of the experimental procedure. (B and C) Micrographs of Neurog2ERT2-infected astrocytes (red) immunostained for GFAP (green) and βIII-tubulin (white), without (B) or with (C) OHT treatment starting at 6 days after being plated. Scale bars, 100 μm. (D) Histogram depicting the proportion of GFAP+ or βIII-tubulin+ cells among infected cells upon delayed Neurog2ERT2 activation at 13 DPI. n = 4 independent experiments. (E and F) Histograms of real-time qPCR (E) and HA-Neurog2ERT2 μChIP-PCR (F) of astrocyte cultures treated as indicated in the legend (early, early OHT treatment, gray bars in E from Figure 1A; and delayed, OHT treatment 6 days later). For (F) cells were exposed to OHT treatment for 24 hr. Percentages of input chromatin were quantified in duplicate from three independent biological samples (mean ± SEM). Significance was tested between samples and respective Dll1 ORF negative region by two-tailed unpaired t test (^∗^p < 0.05, ^∗∗^p < 0.01, ^∗∗∗^p < 0.0001). (G) Scheme of the experiment. (H–J) Micrographs of astrocytes transfected with the constructs indicated on top of the panels with a 5 day delay immune-stained for βIII-tubulin 8 days post transfection (DPT). Scale bars, 50 μm. (K) Histogram depicting the proportion of βIII-tubulin+ cells at 8 DPT. Mean ± SEM; n = 4 independent experiments (^∗^p < 0.05). See also Figure S5.

As astrocytes at postnatal stages are still plastic and proliferate (Ge et al., 2012; Laywell et al., 2000), we tested how reprogramming would be affected if astrocytes were cultured for a longer time. To this end, we maintained Neurog2ERT2-transduced murine astroglial cells in culture for 6 or 8 extra days (data not shown) before starting OHT treatment for 6 days (Figure 5A; condition is referred to as “delayed induction” or prolonged culture [6 days after passaging], while the condition described in Figure 1A is referred to as “early induction” [1–2 days after passaging]). Similar to the untreated controls (Figures 5B and 5D), very few neurons appeared in OHT-treated Neurog2ERT2-transduced prolonged cultures (Figures 5C and 5D) with the majority of them still expressing GFAP and maintaining astroglial morphology. Likewise, delayed induction of Ascl1ERT2 also impaired reprogramming significantly, albeit less dramatically than for Neurog2ERT2 (data not shown). Therefore, prolonged culture of astrocytes renders them more resistant to proneural factor-induced reprogramming, which is similar to previous results obtained by multiple passages of astrocyte cultures (Price et al., 2014).

The expression of the selected downstream targets was examined after delayed induction, and *NeuroD4* was the only target still upregulated by Neurog2ERT2, albeit 5-fold less than it was after early activation of Neurog2ERT2 (Figure 5E). ChIP-qPCR on early OHT-treated Neurog2ERT2-transduced cells revealed that Neurog2ERT2 was significantly enriched at several of its downstream targets (*Atoh8*, *Insm1*, *NeuroD1*, *NeuroD4*, *Prox1*, *Sox11*, and *Trnp1*; Figure 5F, ChIP early), indicating that Neurog2ERT2 directly activates these targets in astroglia by binding to their regulatory elements. However, with the delayed induction protocol, Neurog2ERT2 was bound less to *NeuroD1*, *NeuroD4*, and *Trnp1* promoters (Figure 5F), which is statistically not different from the negative control region (Dll1 ORF). Thus, astroglial cells in culture are not in a stable permissive state for reprogramming but they become increasingly refractory to conversion into neurons, a process that might involve a reduced accessibility of Neurog2 to target genes important for the reprogramming process.

### Selected Target Genes Downstream of Neurog2 and Ascl1 Induce Reprogramming of Prolonged Astroglia Cultures

If the failure of target gene activation is responsible for the low reprogramming efficiency in the prolonged cultures, this should be overcome by expression of these targets (Figure 5G). Indeed, in cultures maintained for a longer time, combinations of ND4 with Insm1, Prox1, or Sox11 elicited the generation of neuronal cells (Figures 5I and 5J) more efficiently than ND4 alone (Figures 5H and 5K). Likewise, combining *Neurog2ERT2* with *Insm1*, *NeuroD4*, *Prox1*, or *Sox11* led to neuronal reprogramming also in prolonged cultures, while cells co-transduced with Neurog2ERT2 and a control virus largely remained astroglia (Figures S5A–S5E, quantification in Figure S5F).

Thus, impairment in neuronal reprogramming in prolonged astroglial cultures is due to a failure in the activation of these common neurogenic fate determinants while the underlying downstream neurogenic program is still amenable for activation.

### REST Represses NeuroD4 Transcription in Competition with Neurog2

**Figure 6:**
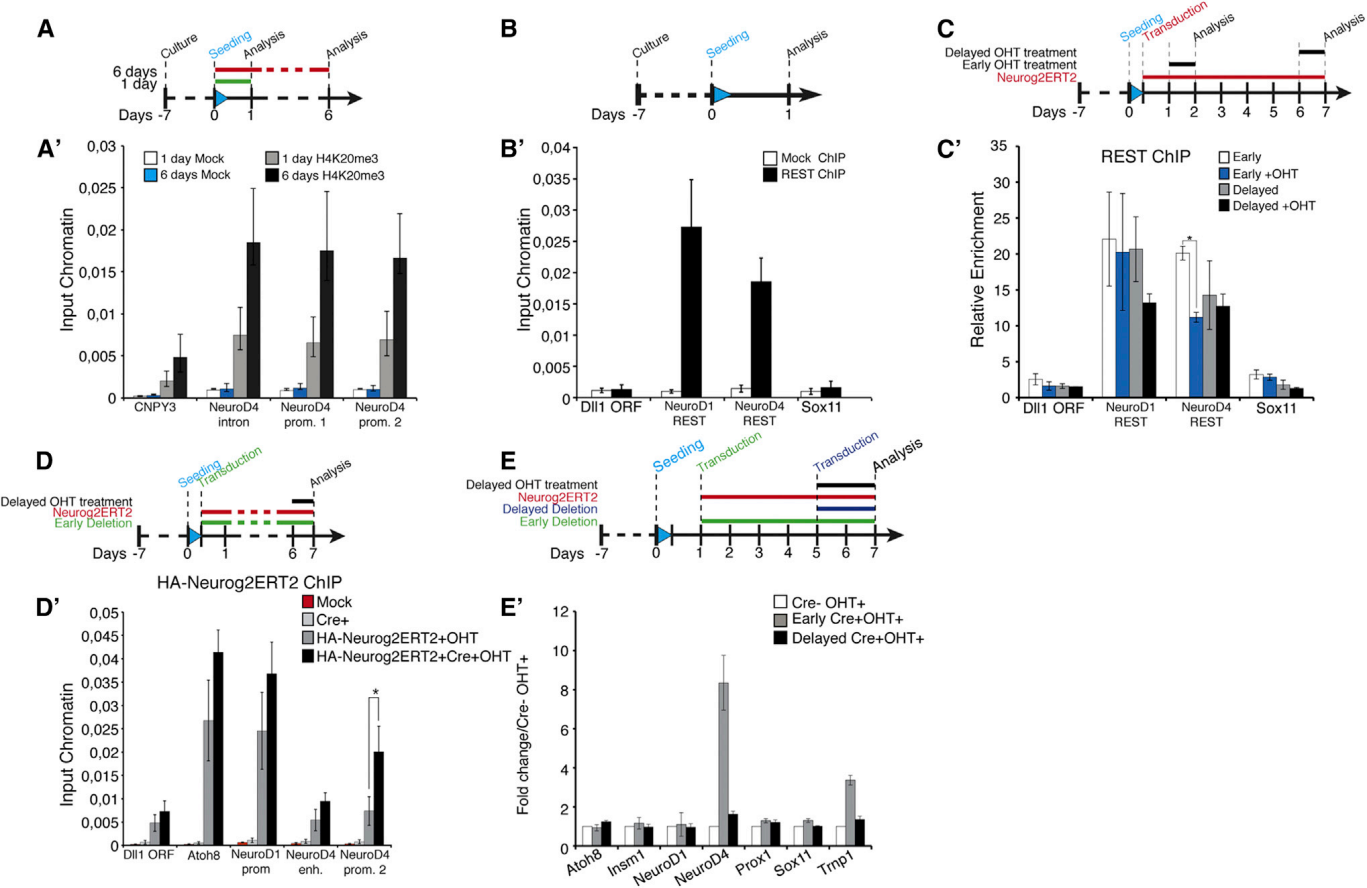
Chromatin Marks and REST Binding at Regulatory Regions of the Downstream Targets NeuroD4, NeuroD1, and Sox11 (A and A′) H4K20me3 μChIP-PCR on immunoprecipitated material from astroglia cultures collected 1 day or 6 days after being plated as indicated in the scheme at top of (A). (B and B′) Analysis of REST binding to *NeuroD4* by μChIP-PCR on immunoprecipitated samples from short-term astroglia cultures as indicated in the scheme in (B). Amplification of the REST binding element within the *NeuroD1* intron was used as a positive control while a region within the promoter of Sox11 was used as a negative control. Percentages of input chromatin were quantified in duplicate from three independent biological samples (mean ± SEM). (C and C′) REST μChIP-PCR on immunoprecipitated samples from Neurog2ERT2-transduced astrocytes cultured for shorter or longer periods and treated with OHT for 24 hr as indicated at the top of (C). REST ChIP values were normalized to their respective mock ChIP values (mean ± SEM in duplicate from three independent biological samples; two-tailed unpaired t test, ^∗^p < 0.05). (D and D′) HA-Neurog2ERT2 μChIP-PCR on immunoprecipitated genomic DNA from delayed astroglia cultures. RESTflox cKO were transduced with Neurog2ERT2 and adeno-Cre virus with a late OHT induction as indicated (D). The *Atoh8* promoter and *NeuroD1* promoter regions were used as controls for the effect of REST deletion on Neurog2 binding. Percentages of input chromatin were quantified in duplicate from three independent biological samples (mean ± SEM; two-tailed unpaired t test, ^∗^p < 0.05). (E and E′) Real-time qPCR analysis on Neurog2ERT2-astrocytes treated with OHT for 48 hr after early or late REST Cre-mediated deletion as indicated at the top of the histogram (E). Control samples (Cre- OHT+) were transduced with adeno null virus 1 day after being seeded at the same time as the delayed Cre sample (adeno-Cre virus, Early Cre+OHT+). In parallel, another set of cells was transduced with adeno-Cre virus 5 days later (Delayed Cre+OHT+). Mean ± SEM in duplicate from three independent culture batches. See also Figure S6.

The reduced Neurog2ERT2 binding to target loci upon delayed activation suggested that changes in the chromatin state might take place at these target loci (see Hirabayashi and Gotoh, 2010 for review). We focused on *NeuroD4* as one of the main target genes mediating the reprogramming activity of Neurog2 and Ascl1 in astroglial cells. Between the cultures collected at different time points, we did not observe any significant change in the repressive marks H3K27me3 and 5mC or the active mark H3K4me3 analyzed by ChIP-qPCR at several locations in this gene, including the Neurog2ERT2-bound enhancer, intron, and promoters (Figures S6A–S6E), while H4K20me3 was enriched in prolonged cultured astrocytes compared to short-term cultures (Figures 6A and 6A′). These data suggest that remodeling of the chromatin at *NeuroD4* locus occurred over time such that it became more heterochromatin-like (Wongtawan et al., 2011).

As a repressor complex might be involved in such a change, we focused on REST, known to repress neuronal gene expression in non-neural cells (Jørgensen et al., 2009). By ChIP-qPCR, REST was confirmed to be present at the *NeuroD1/4* loci in astroglial cells soon after plating (Figures 6B and 6B′) (Gao et al., 2011; Johnson et al., 2008). REST ChIP following early activation of Neurog2ERT2 showed significantly reduced binding onto the *NeuroD4* promoter and less so on the *NeuroD1* or *Sox11* promoters (Figures 6C and 6C′). In contrast, Neurog2ERT2 delayed activation had no effect on REST binding (Figures 6C and 6C′), suggesting that the proneural factor Neurog2 and REST can compete for binding at this site in early cultures, but no longer at later stages. Importantly, western blot analysis revealed that REST protein level was unchanged over time (Figures S6F–S6F′′), thus excluding the possibility that Neurog2 could compete with REST early on but not late because of a higher abundance of REST protein in prolonged cultures.

To directly investigate the role of REST in preventing astroglia reprogramming in prolonged cultures, we generated astroglia cultures from P6 cerebral cortex of mice homozygotes for a new conditional allele of REST (hereafter referred to as REST^flox^, see Experimental Procedures and Figure S6G) and transduced them with a Cre-recombinase-encoding adenovirus either immediately after passaging the astrocytes or with a 5 day delay. In both conditions, REST protein disappeared within 48 hr (Figures S6H–S6H′, black arrow). As Cre-mediated recombination removes exon 2 (Figure S6G) and a truncated form appeared in the western blot (Figure S6H′, empty arrow), we verified that this truncated form has no binding capability (i.e., no significant difference in enrichment between REST-ChIP and mock-ChIP samples, and 5- to 10-fold reduced binding capability compared to REST-expressing astrocytes; Figure S6I, and for comparison, Figure 6B). Thus, Cre-mediated deletion of exon 2 generates a truncated form of REST unable to bind to DNA. Upon REST deletion in short-term cultures, both *NeuroD1* (Gao et al., 2011) and *NeuroD4* were upregulated, while REST ablation in prolonged cultures had no significant effect on *NeuroD1* or *NeuroD4* expression (Figures S6J–S6J′).

To test whether REST could prevent Neurog2ERT2 from binding to the *NeuroD4* promoter in astrocytes cultured for 6 days (Figure 6D), we co-infected the cultures with Cre and Neurog2ERT2, thus deleting REST from the beginning of the culture, and initiated OHT treatment 6 days later. ChIP-qPCR revealed a significant increase of Neurog2ERT2 onto *NeuroD4* promoter compared to REST-expressing cells, with a small effect on Neurog2ERT2 binding to *Atoh8* and *NeuroD1* loci (Figure 6D′). In these conditions (early REST deletion and delayed Neurog2ERT2 activation, Figure 6E), *NeuroD4* and *Trnp1* were upregulated (Figure 6E′, gray bars). However, when REST was removed 5 days after Neurog2ERT2 transduction (Figures 6E and 6E′), *NeuroD4* was not upregulated after Neurog2ERT2 delayed activation (Figure 6E′, black bars).

Together, these data suggest that *NeuroD4* becomes less prone to activation over time, likely through the initial transient repressor activity of REST followed by a histone modification that makes the locus more compact.

### REST Deletion Alleviates the Reprogramming Blockage in Prolonged Astrocytic Cultures

**Figure 7:**
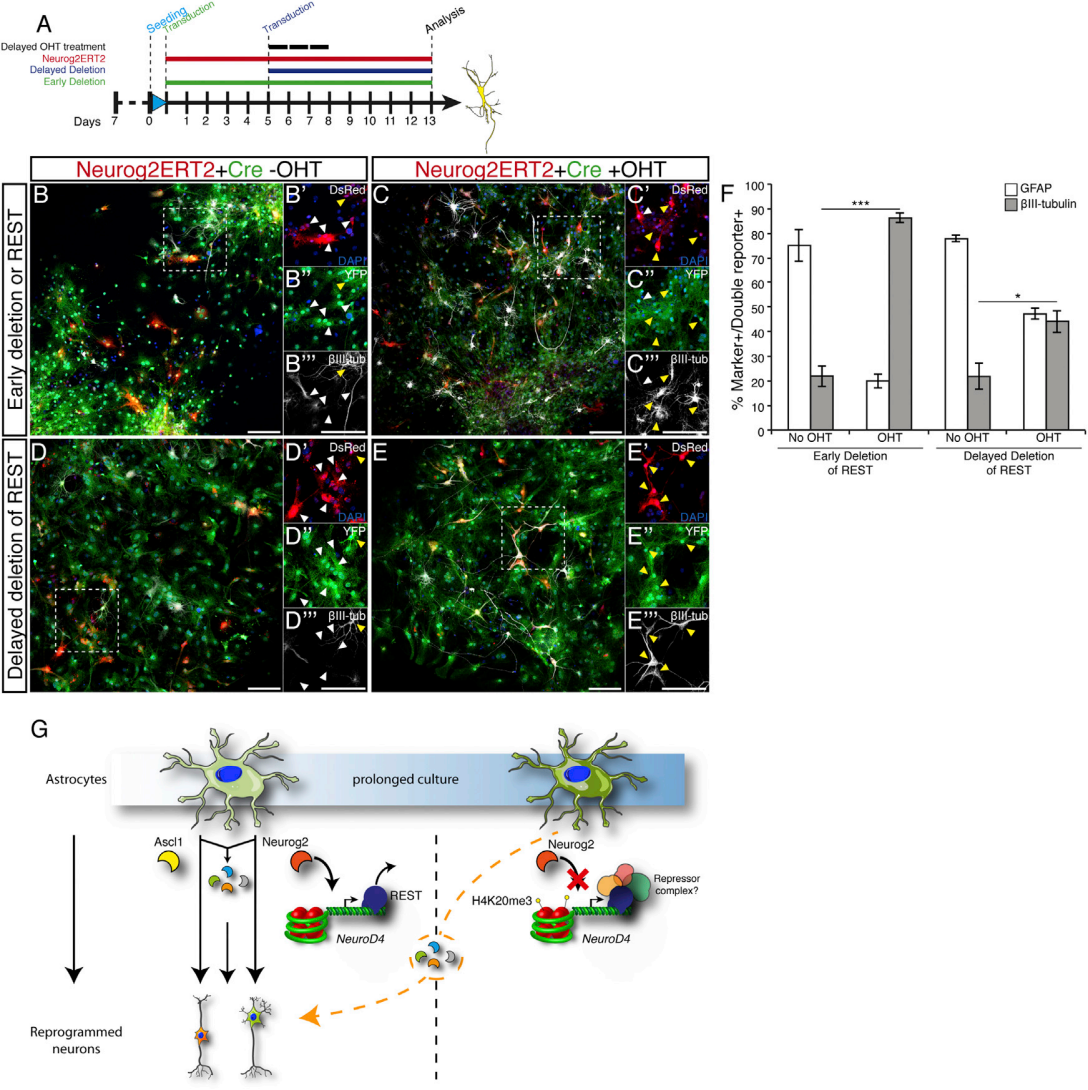
Deletion of REST Removes Reprogramming Block in Astrocytes (A) Schematic representation of the experimental procedure. (B–E) Micrographs of Neurog2ERT2-infected astrocytes (red) with early (B and C) or late (D and E) deletion of REST by infection with a Cre containing viral vector (green) immunostained for the neuronal marker βIII-tubulin (white) at 8 DPI. Yellow arrowheads indicate triple positive cells (DsRed, YFP, βIII-tubulin) while white arrowheads indicate double positive cells (DsRed, GFP). Scale bars, 150 μm. (F) Histogram depicting the proportion of co-transduced double positive cells (red and green) for the astrocytic marker (GFAP, white bars) or the neuronal marker (βIII-tubulin, black bars). Mean ± SEM, three independent biological samples; two-tailed unpaired t test, ^∗^p < 0.05; ^∗∗∗^p < 0.001. (G) Postnatal (day 6–7) mouse cortical astrocytes transduced with Ascl1 or Neurog2 are reprogrammed into neurons. However, when cells are maintained longer in culture, increasing levels of H4K20me3 modify the local chromatin environment that becomes favorable to the repressive complex REST. Consequently, Neurog2 fails to access the NeuroD4 promoter. This is bypassed by common downstream transcription factors to both Ascl1 and Neurog2 that are able to generate neurons also in prolonged astrocytic cultures. Unidentified REST co-factors might be recruited to the locus to further remodel the chromatin over time. See also Figure S7.

To examine the effect of REST deletion on Neurog2ERT2-dependent neuronal conversion, REST^flox^ astrocytes were co-infected with Neurog2ERT2- and Cre-encoding viruses soon after being plated, or with a 5 day delay (Figure 7A). Cultures were then treated for 3 consecutive days with OHT and analyzed 8 DPI (Figures 7B–7E, Figures S7A–S7D). As previously reported (Xue et al., 2013), REST deletion generated a fraction of βIII-tubulin+ cells on its own without Neurog2ERT2 activation (around 20%, Figure 7F); strikingly, however, 90% of Cre/Neurog2ERT2 transduced cells were βIII-tubulin+ after early REST deletion and delayed Neurog2ERT2 activation (Figure 7D). Delayed Cre-mediated REST deletion still allowed 50% of Cre/Neurog2ERT2 double positive cells to convert into βIII-tubulin+ neurons after induction (Figure 7D), suggesting that other mechanisms are gradually taking over to block reprogramming.

Thus, REST is a key factor in silencing main neurogenic targets of proneural factors such that they are no longer accessible for reprogramming in astrocytes in prolonged cultures.

## Discussion

The present study unraveled the transcriptional events taking place in the initial phases of astrocytes converting into neurons. This conversion occurred swiftly, in a dynamic manner, and with very distinct transcriptional programs between the proneural factors Ascl1 and Neurog2. Thus, even within the same cell type from the same brain region maintained in the same environment, forced induction of glutamatergic and GABAergic neuronal fate follows essentially distinct paths, with relatively few genes common to both neurogenic cascades. The analysis of the identified shared target genes led us to identify a particularly important subset of downstream targets capable, when combined, of directly reprograming astrocytes into functional neurons. Among these, *NeuroD4* seems instrumental to force direct reprogramming, and investigating the failure of *NeuroD4* induction in reprogramming-resistant astrocytes led us to uncover an important mechanism of chromatin accessibility control at the *NeuroD4* locus. Indeed, the binding of REST close to the *NeuroD4* promoter prevents the recruitment of Neurog2, while accumulation of H4K20me3 occurred over time. Therefore, this work sheds light on some of the earliest mechanisms decreasing astrocyte reprogramming into neurons.

### Similarities and Differences between Gene Regulation in Development and Direct Reprogramming

Activation of Neurog2ERT2 or Ascl1ERT2 in astroglia cells revealed a highly dynamic regulation of gene expression within the first 48 hr of direct reprogramming: only a small subset of genes (7% and 13.5% for Neurog2 and Ascl1, respectively) was regulated at least at two time points (Figures S1P and S1P′), suggesting a fast and hierarchical sequence of gene regulation. About one-third (188 out of 626) of the genes regulated by Neurog2ERT2 at any time in our analysis are common to the genes regulated by Neurog2 in the developing cerebral cortex in vivo (Gohlke et al., 2008), and similar results were obtained by comparing Ascl1ERT2-regulated genes with Ascl1-electroporated cells in vivo (527 out of 1,669; Gohlke et al., 2008). The proportion of commonly regulated genes is rather low (18% at 48 hr, Figures S7E and S7F) when compared to Ascl1-regulated genes in neuronal reprogramming of MEFs (Wapinski et al., 2013). However, this expression analysis was performed at 48 hr with tetracycline-inducible cells, and, given the fast dynamic regulation of targets observed here, we can only conclude that at least some common target genes are activated during reprogramming of cultured MEFs or astrocytes (Table S7). These are enriched for neuronal differentiation and axon-related genes (Figure S7G, Table S7), such as Dll3, Dcx, neurofilaments, and the known targets Dlx2/3. *Hes6* was the only gene present in all the transcriptome data examined (Neurog2ERT2, Ascl1ERT2, in vivo Neurog2, Ascl1 gain-of-function, Neurog2 loss-of-function (Gohlke et al., 2008), and Ascl1 in MEFs (Wapinski et al., 2013)) with 14 genes present in at least five different analyses (*Arl4A*, *Coro2B*, *Cxadr*, *Dll3*, *Efhd2*, *Gpm6B*, *Hes5*, *Homer2*, *Isl1*, *Lrrc17*, *Plk3*, *Rgs16*, and *Shf*). Thus, even in very different cell types at different developmental stages, some common target genes regulated by these proneural factors emerge.

### Identification of Common Neurogenic Factors

Among the genes regulated by Neurog2 and Ascl1 in astrocyte reprograming, many are pan-neuronal, such as *Elavl2*, synuclein a (*Snca*), *neuronal pentraxin* (*Nptx1*), *D11Bwg0517e* (*Rfox3*, also known as NeuN), and *βIII-tubulin* (*tubb3*), as well as several key neurogenic transcription factors widely expressed in neurogenesis, reflecting their implication in many different neuronal lineages (Figure 1H, Table S6).

Loss-of-function studies on the common downstream targets revealed their crucial role in mediating Neurog2- and Ascl1-induced reprogramming (Figure 2). Interestingly, most of the cells transduced with Neurog2 and specific miRNAs for these targets (Hes6, Insm1, NeuroD4, and Sox11) were negative for both the neuronal marker βIII-tubulin and the astroglial marker GFAP (Figure 2D), suggesting that activation of these targets is not required to block the astroglial fate but rather to induce the neuronal fate. Conversely, the combination of just two of these common neurogenic transcription factors is sufficient to trigger reprogramming of cells into functional neurons from both mouse astroglia (Figures 3, 4, and S3) and human astrocytes and MEFs (Figure S4), suggesting that the identified targets mediate critical biological processes required to induce the neuronal fate, such as transcriptional regulation and cytoskeleton reorganization.

Among the factors tested, NeuroD4 was the only gene capable of reprograming astrocytes into functional neurons on its own. However, only a minority of NeuroD4-induced neurons seem to complete synaptic maturation, while the co-expression of Insm1 seems sufficient to allow them to reach a fully mature synaptic glutamatergic phenotype. Thus, the NeuroD family of bHLH transcription factors (including also NeuroD1 and 2; Guo et al., 2014; Yoo et al., 2011) appears to be particularly important in neuronal reprogramming.

### REST Is a Critical Repressor of NeuroD4

When Neurog2ERT2-transduced astrocytes were maintained in culture for 6 days before OHT treatment, only a small fraction of them converted into neurons, most likely as a consequence of the reduced induction of some targets, such as *NeuroD4* (Figure 4E), suggesting that within this short period of time reprogramming blocks were already established.

Examining the chromatin landscape changes at the *NeuroD4* locus, we detected an enrichment of the heterochromatin-associated histone mark H4K20me3 (Wongtawan et al., 2011) at the *NeuroD4* promoter in prolonged cultured cells, suggesting a progressive reduction of chromatin accessibility at this locus. Interestingly, REST is highly enriched at both *NeuroD1* and *NeuroD4* loci initially but less at the *NeuroD4* promoter in astrocytes cultured for 6 more days (Figure 6C′), suggesting that REST is important in initiating the silencing of *NeuroD4*, but additional repressive mechanisms may be involved at later stages. Consistent with this hypothesis, the binding competition between Neurog2 and REST only occurred in cultures soon after they were plated (Figure 6C′), and a strong activation of *NeuroD4* in prolonged cultures occurred only upon early deletion of REST (Figure 6E). These observations thus revealed a temporal window during which REST binding/activity can be modulated.

REST ablation resulted in a striking improvement of reprogramming efficiency upon delayed Neurog2ERT2 activation when REST was deleted early but also when it was deleted late, thus suggesting important functions of REST-regulated genes other than NeuroD4 in astrocyte reprogramming. Further studies will be required to examine the mechanism underlying the essential role of REST in orchestrating gene silencing in astrocytes (Figure 7G). In different cell types, recruitment of other factors, such as HP-1 or HDAC1, is important to further silence gene transcription. However, we did not observe a significant difference in HP-1 or HDAC1 binding to NeuroD4 between short- and prolonged astroglia cultures (data not shown). REST has recently been implicated in PTB-regulated miRNA-based MEF reprogramming (Xue et al., 2013) and identification of specific co-factors/regulators needs to be explored in astrocytes.

Importantly, our results revealed a hierarchical mode of target gene blockage mediating alternative fates. While Neurog2 could no longer regulate some of its targets, such as *NeuroD4*, in prolonged cultures, the targets of NeuroD4 are still accessible, since NeuroD4 with or without an additional common factor could still mediate reprogramming as efficiently as in short-term cultured astrocytes. Thus, our data suggest a developmental hierarchy in shutting off genes of alternative fates, a novel concept in elucidating the hurdles for direct reprogramming.

## Experimental Procedures

### Cell Cultures of Astroglia from the Postnatal Mouse Cerebral Cortex

Astrocytes were cultured as previously described (Heinrich et al., 2010; Heins et al., 2002). MEFs were isolated as described (Vierbuchen et al., 2010). Human astrocytes were purchased from ScienCell (cat. #1800) and expanded as described in the protocol. For details on cell culture see the Supplemental Experimental Procedures.

### Immunocytochemistry

Cells were fixed and stained as previously described (Heinrich et al., 2011). For details and antibodies used see the Supplemental Experimental Procedures.

### RNA Extraction and Real-Time qPCR

6 to 12 wells from 24-well plates were collected for each time point. Subsequently, RNA was extracted with the RNeasy Plus Micro Kit (QIAGEN) according to the manufacturer’s instructions, and genomic DNA was removed. RNA was retro-transcribed with SuperScriptIII Reverse Transcriptase and Random Primers (Roche). Each cDNA sample was diluted 1:5 and 1 μl was used for each quantitative real-time reaction. Real-time qPCR was performed on a LightCycler480 instrument (Roche) with the LightCycler Probe Master kit (Roche) and Monocolor Hydrolysis Probe (UPL) Probe (Roche) according to the manufacturer’s instructions (20 μl final volume). The expression of each gene was analyzed in triplicate. Data were processed with the ΔΔCt method (Livak and Schmittgen, 2001). Quantification was performed on three independent samples. Primers and probes are listed in the Supplemental Experimental Procedures.

### Microarray Analysis

10 μg of amplified antisense RNA (aRNA) was hybridized on Affymetrix Mouse Genome 430 2.0 arrays containing about 45,000 probesets. Staining and scanning was done according to the Affymetrix expression protocol. GO term analysis was performed using DAVID (http://david.abcc.ncifcrf.gov). For details see Supplemental Experimental Procedures.

### Plasmids and DNA constructs

cDNA of selected genes was subcloned into self-inactivating retroviral vectors containing the actin promoter with cytomegalovirus enhancer (pCAG) driving the expression of the genes of interest linked to a fluorescent reporter through internal ribosomal entry site (IRES) as previously described (Heinrich et al., 2011). Flag-HA-Ascl1ERT2 and Flag-HA-Neurog2ERT2 were obtained by a fusion of the transcription factor cDNA together with the ERT2 domain of the estrogen receptor. For ChIP experiments, DsRed cDNA present in pCAG-Neurog2ERT2-IRES-DsRed was replaced with Puromycin cDNA to allow cell selection in the culture. For details see the Supplemental Experimental Procedures.

### Micro-ChIP and qPCR

Around 100,000 cells per sample were used for micro-ChIP (μChIP). For details see the Supplemental Experimental Procedures.

### Western Blot

Cells were washed three times with 1X cold PBS, lysed with urea buffer (8M urea, 1M thiourea, 0.5% [w/v] CHAPS, 50mM DTT, and 24mM spermine), scraped with a sterile disposable cell scraper (Costar), transferred to an Eppendorf tube, and centrifuged at 14,000 rpm at room temperature for 30 min. Equal amounts of protein were loaded onto polyacrilamide gels (Novex, Life Technologies) and blotted with anti-REST (1/200; Millipore, 07-579) or anti-LaminB (1/1000; Santa Cruz, sc-6216 and sc-6217).

### Patch-Clamp Recording

Whole-cell current-clamp recordings were made using an npi ELC-03XS amplifier (npi, Tamm, Germany), which allowed current-clamp recordings in bridge mode and voltage-clamp measurements. For further information, see the Supplemental Experimental Procedures.

### Conditional REST Mouse Line

Mouse ESCs targeted with the L1L2_Bact_P cassette were obtained from the Sanger EUCOMM project (clone EPD0105_1_E05, http://www.informatics.jax.org/allele/key/609045) and injected into blastocysts to generate heterozygous animals with loxP sites flanking the second exon of REST. Subsequent crossings with Rosa26-floxed stop-YFP reporter mice (Srinivas et al., 2001) were performed to generate a homozygous REST^flox^/R26YFP line. In order to remove the neo selection cassette from the REST locus, we crossed REST^neoflox^ animals with the Flip recombinase mouse line. Experiments conducted with REST^flox^ mice were performed in accordance with a UK Home Office Project License and approved by the local ethics committee. All animal procedures were carried out in accordance with the policies of the use of animals and human material of the EU and the institutional animal committees implementing them.

## Author Contributions

G.M. collected samples for the microarrays, generated the constructs for gain-of-function and loss-of-function experiments in astrocytes, performed most of the reprogramming experiments on mouse and human astrocytes and MEFs, and analyzed the data. S.G. performed the experiments related to the molecular mechanisms, analyzed the reprogramming in REST^flox^ mice, and analyzed the data. G.M., S.G., B.B., F.G., and M.G. conceived the experimental procedures; G.M., S.G., F.G., and M.G. wrote the manuscript; D.D. generated the initial inducible constructs; H.F.J. generated the REST^flox^ line; M.I. and J.B. performed the microarrays and analyzed the microarray data; B.S. performed all of the patch-clamp recordings; and S.S. and F.T. analyzed RNA-seq results and compared them with the microarray results.

## Accession Numbers

Array data have been submitted to GEO under the accession number GEO: GSE60389.

## Supplemental Information

Document S1. Figures S1–S7, Table S5, and Supplemental Experimental Procedures

Table S1. Overview of Regulated Genes upon Neurog2ERT2 Induction

Table S2. Gene Ontology Terms Associated with Genes Regulated by Neurog2ERT2 after 4 hr of OHT Treatment Analysis was performed using the DAVID analysis tool. Cut off, p < 0.05.

Table S3. Overview of Regulated Genes upon Ascl1ERT2 Induction

Table S4. Gene Ontology Terms Associated with Genes Regulated by Ascl1ERT2 after 4 hr of OHT Treatment Analysis was performed using the DAVID analysis tool. Cut off, p < 0.05.

Table S6. Overview of Probesets Regulated by Neurog2ERT2 and Ascl1ERT2 after 24 of OHT Treatment Pan-neuronal genes are indicated in red, genes associated with neurons with some regional differences are indicated in orange, or genes not directly associated with neuronal expression are indicated in black.

Table S7. Overview of Genes Commonly Regulated by Ascl1ERT2 after OHT at Any Time and Genes Regulated by Ascl1 in Wapinski et al. (2013) Genes in red are associated with nervous system expression, genes in blue are associated with neuronal expression, and genes in black genes are not directly related to neuronal expression.

Document S2. Article plus Supplemental Information
