# Supplementary material for: Transcriptional Mechanisms of Proneural Factors and REST in Regulating Neuronal Reprogramming of Astrocytes
Source: Cell Stem Cell. 2015 Jul 2;17(1):74–88. doi: 10.1016/j.stem.2015.05.014 (PMC4509553; doi:10.1016/j.stem.2015.05.014)
Supplement: Document S1. Figures S1–S7, Table S5, and Supplemental Experimental Procedures [file mmc1.pdf]

Cell Stem Cell
Supplemental Information

# Transcriptional Mechanisms of Proneural Factors and REST in Regulating Neuronal Reprogramming of Astrocytes

Giacomo Masserdotti, Sébastien Gillotin, Bernd Sutor, Daniela Drechsel, Martin Irmler, Helle F. Jørgensen, Steffen Sass, Fabian J. Theis, Johannes Beckers, Benedikt Berninger, François Guillemot, and Magdalena Götz

# Figure S1

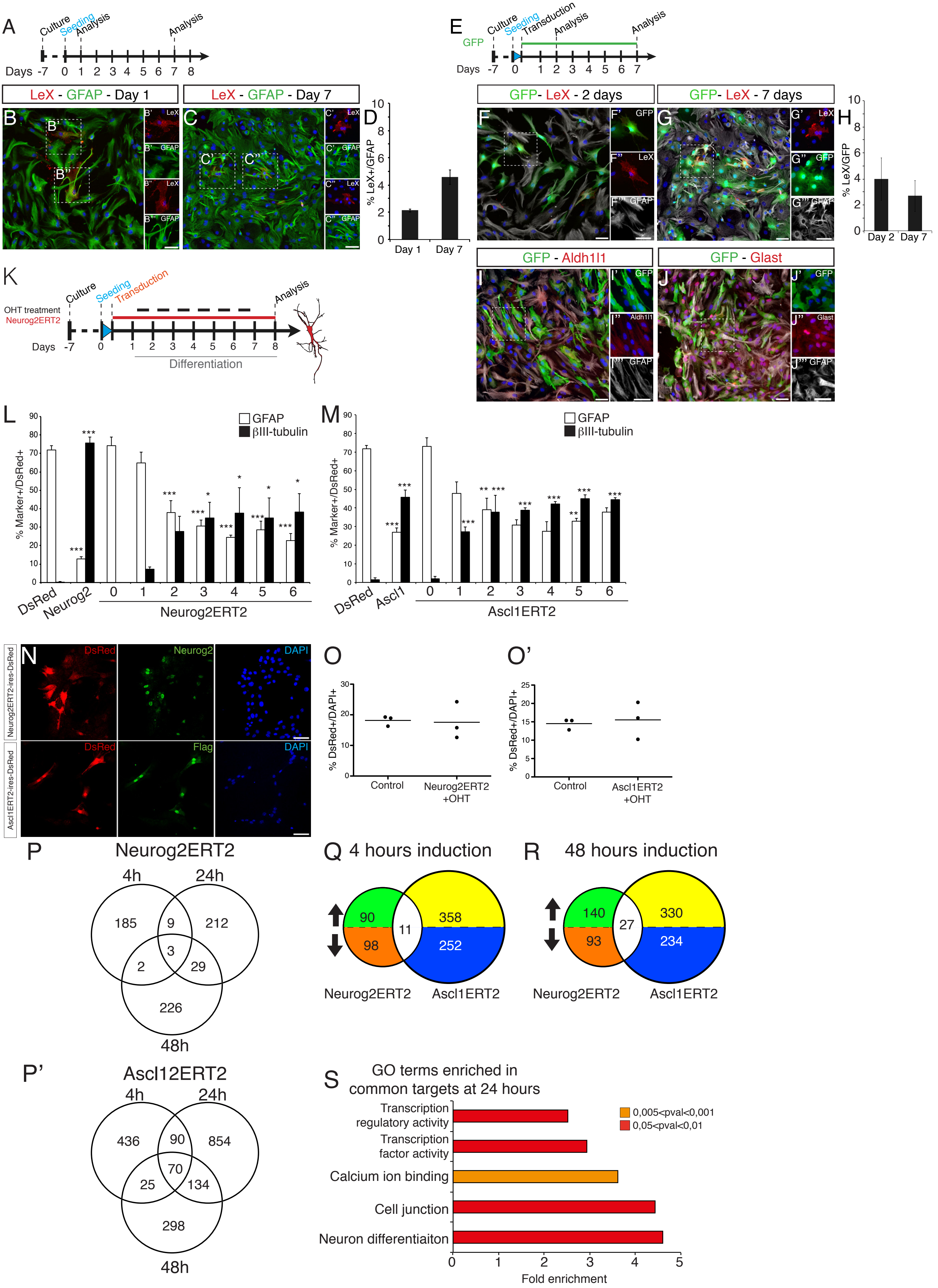
A
Culture
Seeding
Analysis
Analysis
Days -7 0 1 2 3 4 5 6 7 8
LeX - GFAP - Day 1
LeX - GFAP - Day 7
% LeX+/GFAP
Day 1
Day 7
E
GFP
Culture
Seeding
Transduction
Analysis
Analysis
Days -7 0 1 2 3 4 5 6 7
GFP- LeX - 2 days
GFP- LeX - 7 days
% LeX/GFP
Day 2
Day 7
GFP - Aldh1l1
GFP - Glast
K
OHT treatment
Neurog2ERT2
Culture
Seeding
Transduction
Analysis
Days -7 0 1 2 3 4 5 6 7 8
Differentiation
L
% Marker+/DsRed+
GFAP
βIII-tubulin
DsRed
Neurog2
Neurog2ERT2
M
Ascl1
Ascl1ERT2
N
DsRed
Neurog2
DAPI
Flag
Neurog2ERT2-ires-DsRed
Ascl1ERT2-ires-DsRed
O
% DsRed+/DAPI+
Control
Neurog2ERT2 +OHT
O'
Ascl1ERT2 +OHT
P
Neurog2ERT2
4h
24h
48h
185
9
212
3
2
29
226
Q 4 hours induction
90
11
358
98
252
Neurog2ERT2
Ascl1ERT2
R 48 hours induction
140
27
330
93
234
P'
Ascl12ERT2
436
90
854
70
25
134
298
S
GO terms enriched in common targets at 24 hours
Transcription regulatory activity
Transcription factor activity
Calcium ion binding
Cell junction
Neuron differentiaiton
0,005<pval<0,001
0,05<pval<0,01
Fold enrichment

# Figure S2

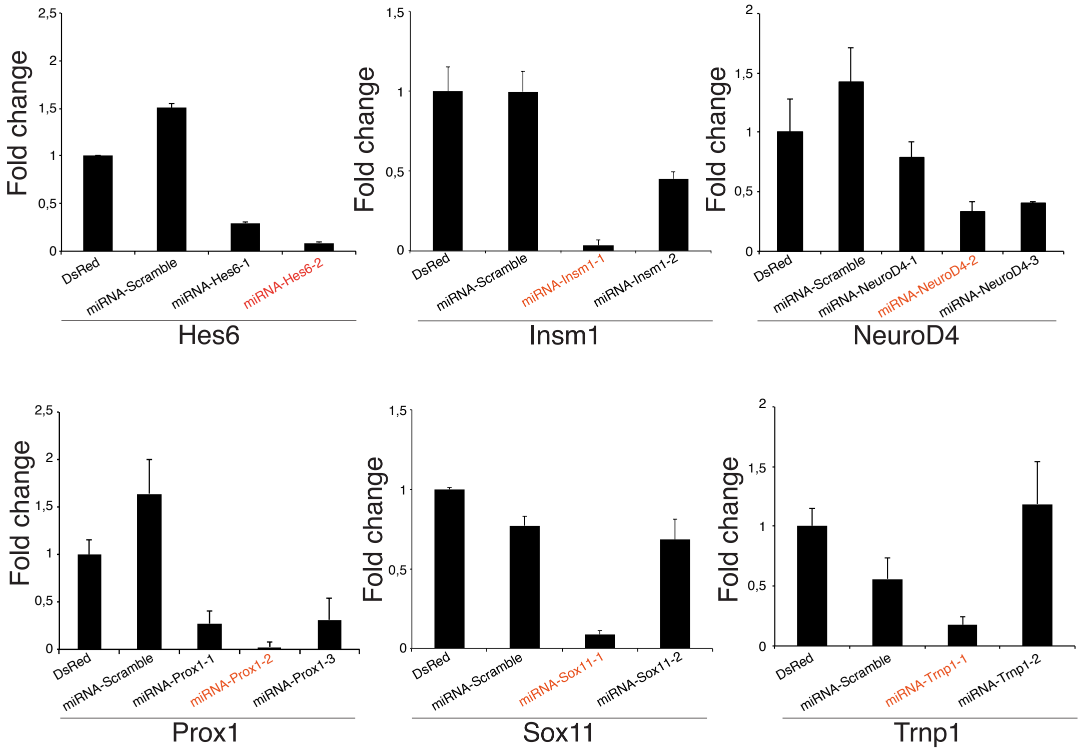

Fold change
DsRed
miRNA-Scramble
miRNA-Hes6-1
miRNA-Hes6-2
Hes6
miRNA-Insm1-1
miRNA-Insm1-2
Insm1
miRNA-NeuroD4-1
miRNA-NeuroD4-2
miRNA-NeuroD4-3
NeuroD4
miRNA-Prox1-1
miRNA-Prox1-2
miRNA-Prox1-3
Prox1
miRNA-Sox11-1
miRNA-Sox11-2
Sox11
miRNA-Trnp1-1
miRNA-Trnp1-2
Trnp1

# Figure S3

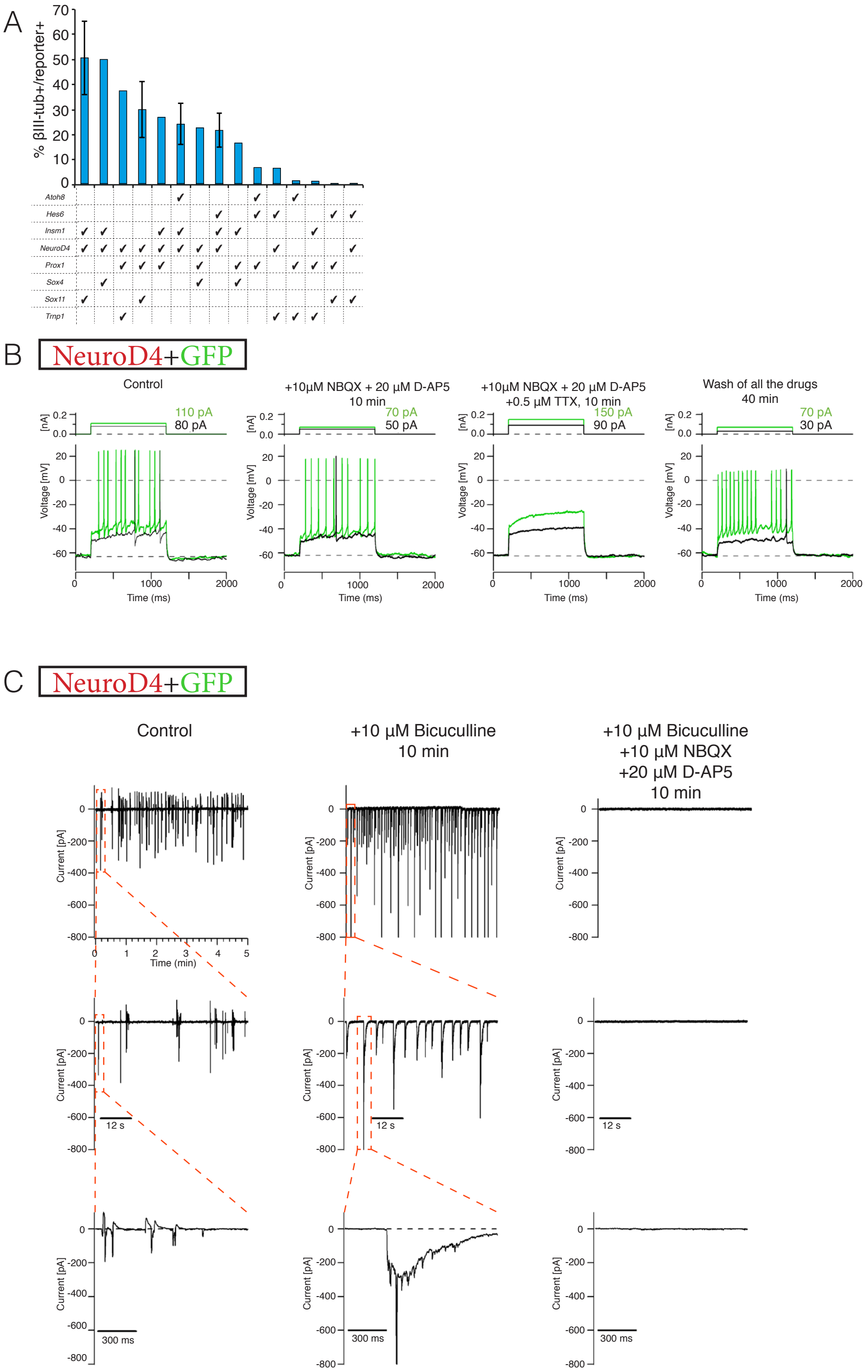

A
% βIII-tub+/reporter+
70
60
50
40
30
20
10
0
Atoh8
Hes6
Insm1
NeuroD4
Prox1
Sox4
Sox11
Tmp1
B
NeuroD4+GFP
Control
+10μM NBQX + 20 μM D-AP5
10 min
+10μM NBQX + 20 μM D-AP5
+0.5 μM TTX, 10 min
Wash of all the drugs
40 min
[nA]
0.2
0.0
110 pA
80 pA
70 pA
50 pA
150 pA
90 pA
70 pA
30 pA
Voltage [mV]
20
0
-20
-40
-60
0
1000
2000
Time (ms)
C
NeuroD4+GFP
Control
+10 μM Bicuculline
10 min
+10 μM Bicuculline
+10 μM NBQX
+20 μM D-AP5
10 min
Current [pA]
0
-200
-400
-600
-800
0
1
2
3
4
5
Time (min)
12 s
300 ms

# Figure S4

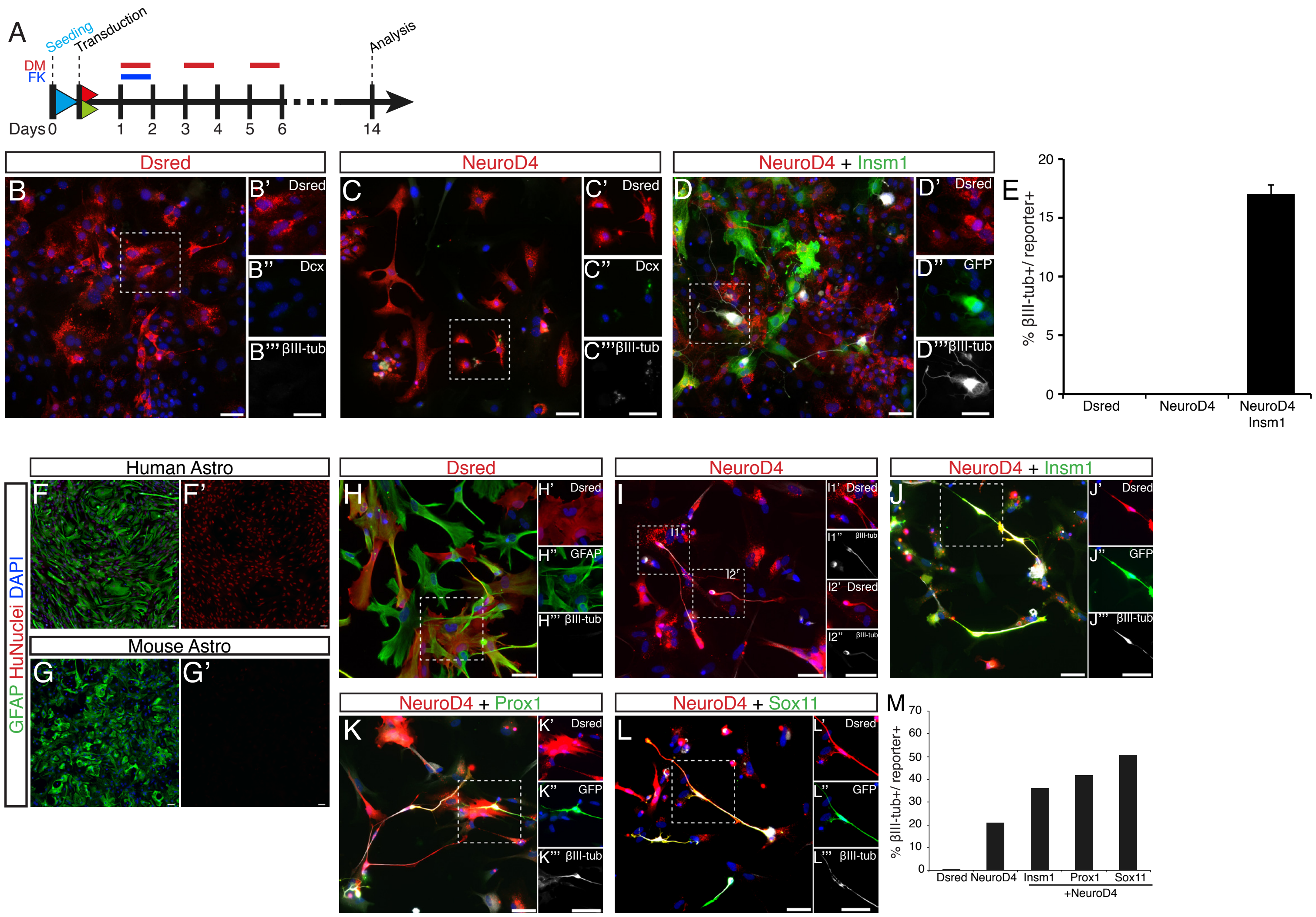
A
Seeding
Transduction
Analysis
DM
FK
Days 0 1 2 3 4 5 6 14
Dsred
NeuroD4
NeuroD4 + Insm1
B B' Dsred B'' Dcx B''' βIII-tub
C C' Dsred C'' Dcx C''' βIII-tub
D D' Dsred D'' GFP D''' βIII-tub
E
% βIII-tub+/ reporter+
Dsred
NeuroD4
NeuroD4 Insm1
GFAP HuNuclei DAPI
Human Astro
Mouse Astro
F F'
G G'
Dsred
H H' Dsred H'' GFAP H''' βIII-tub
NeuroD4
I I1' Dsred I1'' βIII-tub I2' Dsred I2'' βIII-tub
NeuroD4 + Insm1
J J' Dsred J'' GFP J''' βIII-tub
NeuroD4 + Prox1
K K' Dsred K'' GFP K''' βIII-tub
NeuroD4 + Sox11
L L' Dsred L'' GFP L''' βIII-tub
M
% βIII-tub+/ reporter+
Dsred NeuroD4 Insm1 Prox1 Sox11
+NeuroD4

# Figure S5

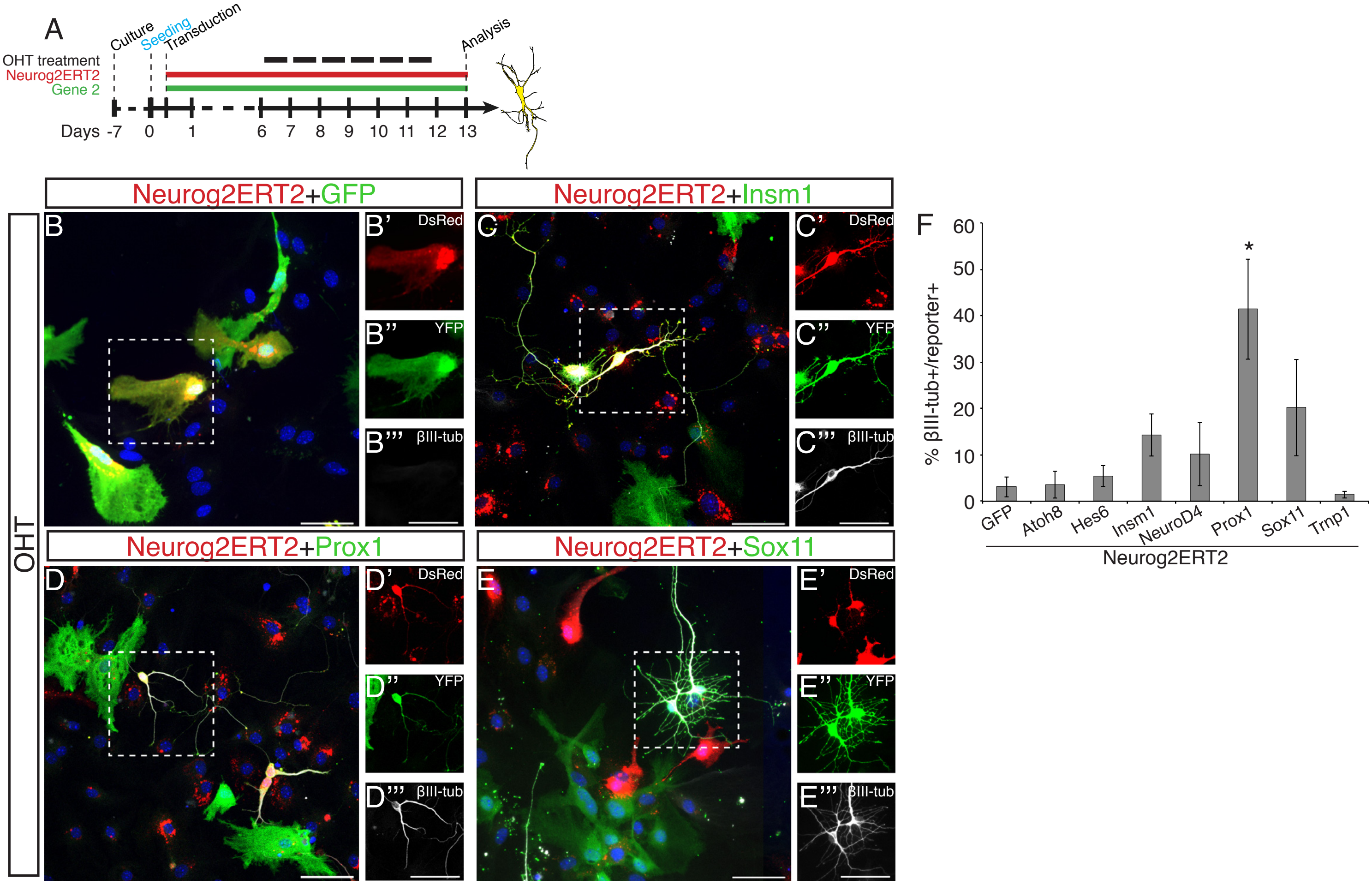

A
OHT treatment
Neurog2ERT2
Gene 2
Culture
Seeding
Transduction
Analysis
Days -7 0 1 6 7 8 9 10 11 12 13
OHT
B
Neurog2ERT2+GFP
B' DsRed
B'' YFP
B''' βIII-tub
C
Neurog2ERT2+Insm1
C' DsRed
C'' YFP
C''' βIII-tub
D
Neurog2ERT2+Prox1
D' DsRed
D'' YFP
D''' βIII-tub
E
Neurog2ERT2+Sox11
E' DsRed
E'' YFP
E''' βIII-tub
F
% βIII-tub+/reporter+
0
10
20
30
40
50
60
*
GFP
Atoh8
Hes6
Insm1
NeuroD4
Prox1
Sox11
Trnp1
Neurog2ERT2

# Figure S6

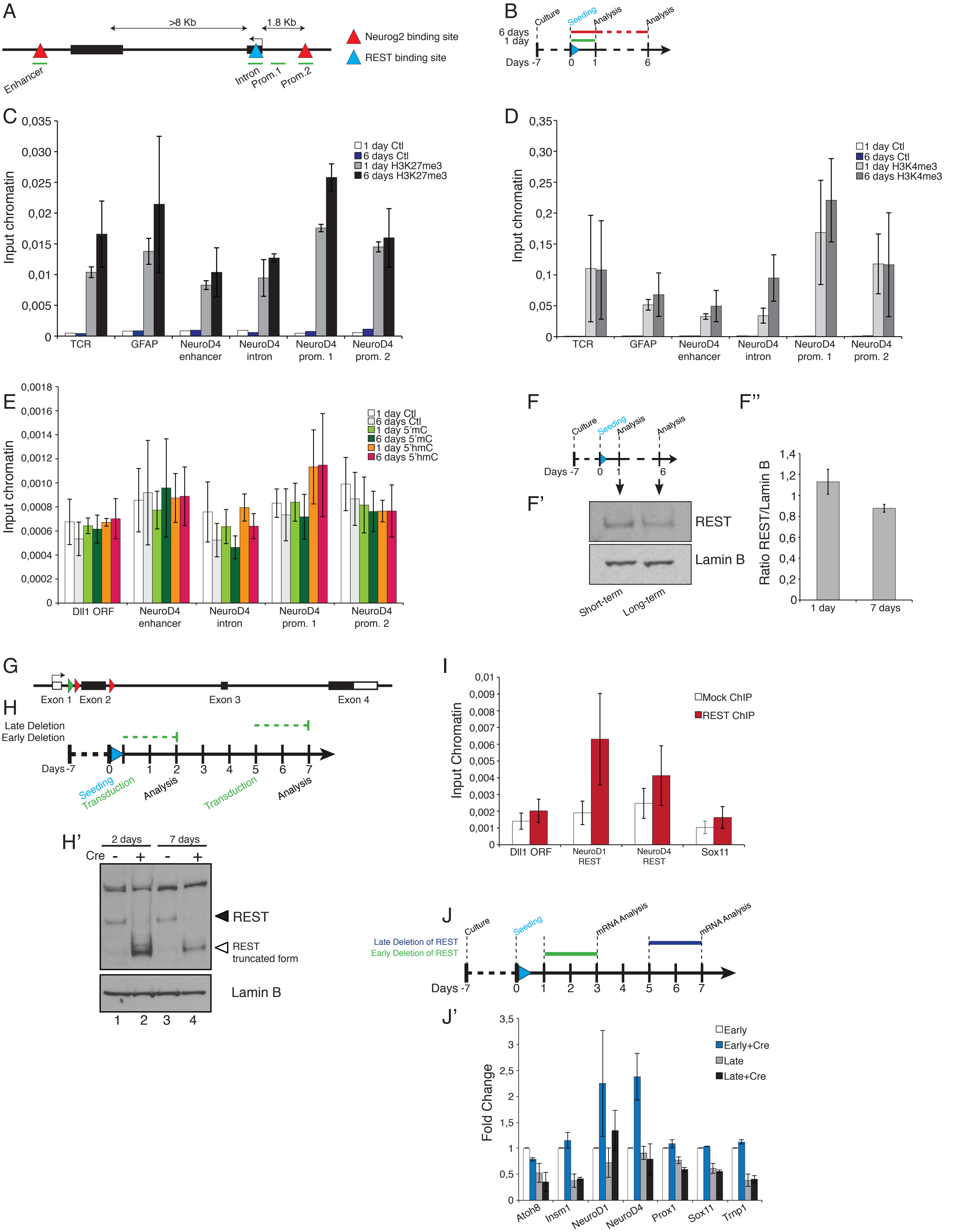

A
>8 Kb
1.8 Kb
Neurog2 binding site
REST binding site
Enhancer
Intron
Prom.1
Prom.2
B
Culture
Seeding
Analysis
Analysis
6 days
1 day
Days -7
0
1
6
C
Input chromatin
1 day Ctl
6 days Ctl
1 day H3K27me3
6 days H3K27me3
TCR
GFAP
NeuroD4 enhancer
NeuroD4 intron
NeuroD4 prom. 1
NeuroD4 prom. 2
D
Input chromatin
1 day Ctl
6 days Ctl
1 day H3K4me3
6 days H3K4me3
E
Input chromatin
1 day Ctl
6 days Ctl
1 day 5'mC
6 days 5'mC
1 day 5'hmC
6 days 5'hmC
Dll1 ORF
F
F'
REST
Lamin B
Short-term
Long-term
F''
Ratio REST/Lamin B
1 day
7 days
G
Exon 1
Exon 2
Exon 3
Exon 4
H
Late Deletion
Early Deletion
Transduction
H'
2 days
7 days
Cre
REST
REST truncated form
I
Input Chromatin
Mock ChIP
REST ChIP
NeuroD1 REST
NeuroD4 REST
Sox11
J
Late Deletion of REST
Early Deletion of REST
mRNA Analysis
J'
Fold Change
Early
Early+Cre
Late
Late+Cre
Atoh8
Insm1
NeuroD1
NeuroD4
Prox1
Sox11
Trnp1

# Figure S7

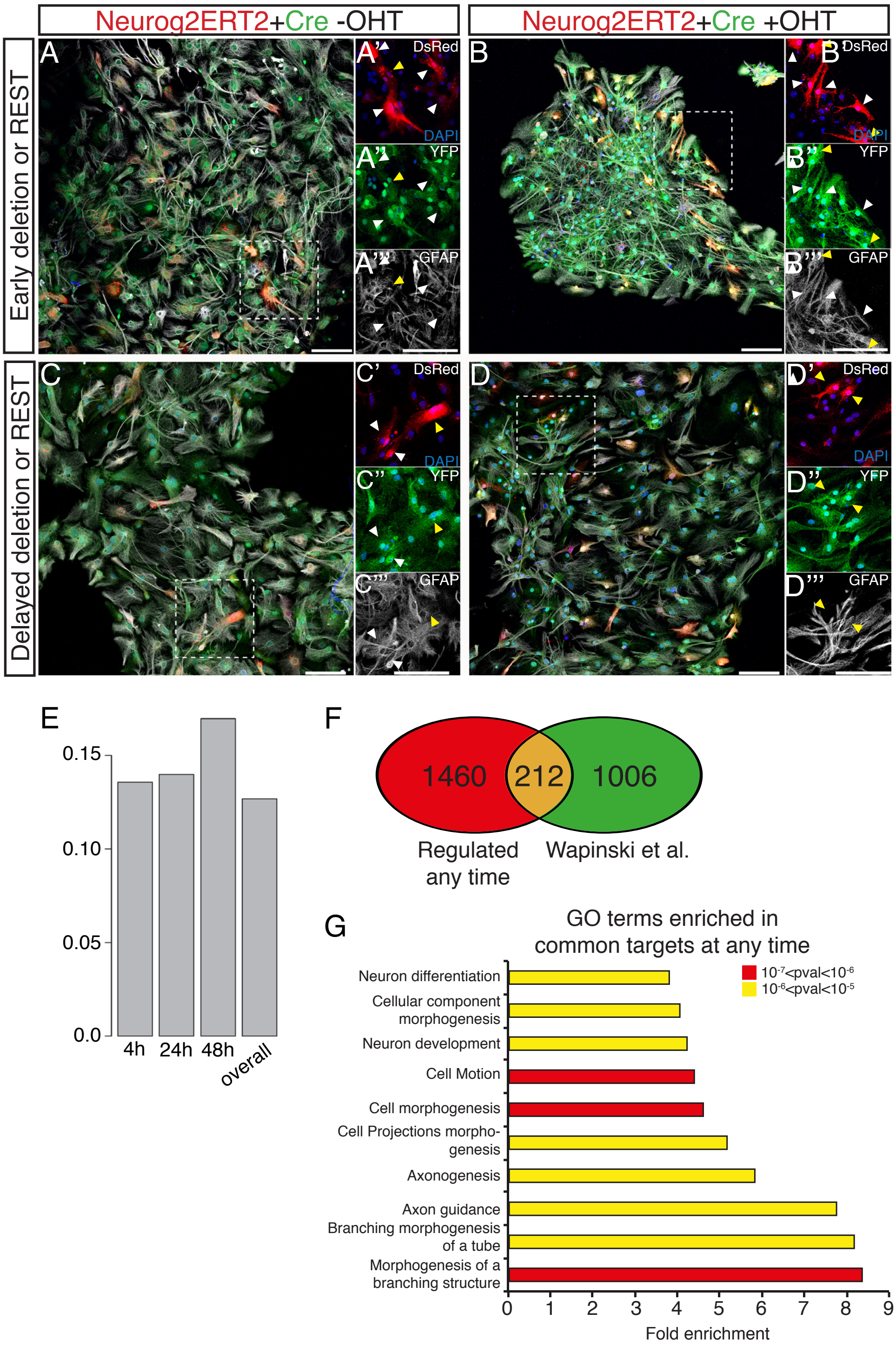
Neurog2ERT2+Cre -OHT
Neurog2ERT2+Cre +OHT
Early deletion or REST
Delayed deletion or REST
A
A'
A''
A'''
B
B'
B''
B'''
C
C'
C''
C'''
D
D'
D''
D'''
DsRed
DAPI
YFP
GFAP
E
0.15
0.10
0.05
0.0
4h
24h
48h
overall
F
1460
212
1006
Regulated any time
Wapinski et al.
G
GO terms enriched in common targets at any time
10-7<pval<10-6
10-6<pval<10-5
Neuron differentiation
Cellular component morphogenesis
Neuron development
Cell Motion
Cell morphogenesis
Cell Projections morphogenesis
Axonogenesis
Axon guidance
Branching morphogenesis of a tube
Morphogenesis of a branching structure
0
1
2
3
4
5
6
7
8
9
Fold enrichment

**Supplementary Figure legends**

*Figure S1. Related to Figure 1*

*Inducible reprogramming and genome-wide expression analysis*

(A) Schematic drawing of the experimental design for Figure S1B-C.

(B,C) Micrographs of astrocytes 1 day (B) and 7 days (C) after plating immune-positive for LeX (red) and GFAP (green). Scale bar: 100$\mu$m.

(D) Histogram showing the proportion of LeX+ cells/GFAP+ in astrocytic cultures at day 1 and day 7. (n=3 independent experiment).

(E) Schematic drawing of the experimental design for Fig. S1F-J.

(F-J) Micrograph of GFP-transduced astrocytes at day 2 (F, I, J) and day 7 (G) immunoreactive for LeX (F, G, red), GFAP (F-J, white), Aldh1l1 (I, red) or Glast (J, red).

(H) Histogram showing the proportion of LeX+/GFP+ cells in astrocytic cultures at day 1 and day 7. (n=4 independent experiment).

(K) Schematic drawing of the experimental design.

(L, M) Histograms depicting the proportion of GFAP+ astrocytes and βIII-tubulin+ neurons after transduction with the constructs indicated and OHT treatment as indicated by the black bar below the histograms. Mean ± SEM; n=4 independent experiments; Statistical test ANOVA and Bonferroni's multiple Comparison Test (*=p<0.05; ***=p<0.001).

(N) Micrographs depicting astrocytes infected with the constructs indicated on the right side of the panels and immunostained for Neurog2 (green in middle panel of the upper row) or the flag-tag of Ascl1ERT2 (green in middle panel of the lower row) in DsRed-expressing cells at 24 hours after OHT treatment at 2 days post infection. Scale bar: 100$\mu$m.

(O-O') Quantification of transduction efficiency in Neurog2ERT2-expressing cells (F), or Ascl1ERT2-transduced cells (F') in absence or presence of OHT for 24 hours.

(P-P') Venn diagrams showing the number of genes significantly regulated in Neurog2ERT2 (P) or Ascl1ERT2 (P') at the time points indicated in the diagrams.

(Q,R) Venn diagram showing the number of genes significantly regulated by Neurog2ERT2 and Ascl1ERT2 at 4 hours (Q) and 48 hours (R) following OHT treatment.

(S) Gene Ontology (GO) terms enriched in the genes regulated by both Neurog2ERT2 and Ascl1ERT2 at 24 hours following OHT treatment.

*Figure S2. Related to Figure 2*

*Validation of microRNA (miRNA)*

Histograms depicting the fold change in expression determined by Real Time qPCR with RNA extracted from cells transfected with the indicated constructs. In red the results obtained with the microRNA selected for further experiments (Figure 2).

*Figure S3. Related to Figure 3 and 4*

*Combinations of downstream targets and characterization of ND4-reprogrammed neurons co-cultured with E14.5 neurons.*

(A) Histogram depicting the proportion of βIII-tubulin+ neurons amongst the astrocytes co-transduced with the plasmids indicated on the x-axis in 2-6 independent culture batches depending on the combinations.

(B) Electrophysiological recordings of ND4-reprogrammed neurons at 28-25 DPI. Lack of effects of NBQX and D-AP5 on action potentials (second trace), while addition of TTX blocked the spike induction (third trace) in a reversible manner (fourth trace) (n=3 cells recorded).

(C) Effect of bicuculline addition (middle traces) and NBQX and D-AP5 (right traces) on the reprogrammed neurons. Note that hyperpolarizing events or outward currents recorded under voltage-clamp conditions at -60mV could be inhibited by the $GABA_A$ receptor antagonist bicuculline. Moreover, the AMPA-receptor antagonist NBQX and the NMDA-receptor antagonist D-AP5 could block the synaptic potentials or currents.

*Figure S4. Related to Figure 4*

*Combinations of downstream targets reprogram murine embryo fibroblasts and human astrocytes*

(A) Schematic drawings of the experiment design for Fig. S4B-E.

(B-D) Example of MEFs transduced with Control (B) NeuroD4 (C) and NeuroD4 and Insm1 (D). Note that only NeuroD4+Insm1 gave rise to βIII-tubulin+ (D, D''') cells at 14DPI.

(E) Histogram depicting the proportion of βIII-tubulin+ cells among reporter positive cells, (n=3 independent experiment).

(F,G) Human astrocytes are positive for GFAP (G) and the human nuclei marker (F') while mouse astrocytes only for GFAP (G, G'). Scale bar: 100µm.

(H-L) Examples of human astrocytes transduced with retrovirus encoding the indicated genes. Scale bar: 100µm.

(M) Histrogram depicting the proportion of βIII-tubulin+ cells over the transduced ones. (n=2 independent cultures).

*Figure S5. Related to Figure 5.*

*Expression of downstream targets together with Neurog2ERT2 rescues reprogramming upon Neurog2ERT2 delayed induction.*

(A) Schematic drawing of the co-transduction of Neurog2ERT2 (red) and a second gene (green) followed by delayed activation of Neurog2ERT2.

(B-E) Examples of astrocytes transduced with the Neurog2ERT2 and the indicated factors and analysed 7 days after the first induction. Inserts show that the cells are DsRed+ (B', C', D', E'), GFP+ (B'', C'', D'', E'') and βIII-tubulin+ (B''', C''', (D''', E'''). Scale bars= 100$\mu$m.

(F) Histogram depicting the proportion of βIII-tubulin+ neurons among DsRed/GFP double+ co-transduced cells at 7 days post differentiation. Mean ± SEM; n=3 independent experiments; (*=$p<0.05$).

*Figure S6. Related to Figure 6.*

*Epigenetic marks at NeuroD4 regulatory regions and analysis of REST expression and function.*

(A) Schematic representation of the mouse *Neurod4* locus. Red and blue triangles represent Neurog2 and REST binding sites respectively and black squares represent exons. Green lines indicate positions of primers used in real time qPCR.

(B) Timeline of sample collection for µChIP analysis.

(C) H3K27me3 µChIP-PCR on immunoprecipitated material from astroglia collected 1 day or 6 days after plating.

(D) H3K4me3 µChIP-PCR on immunoprecipitated material from astroglia collected 1 day or 6 days after plating.

(E) 5'mC and 5'hmC µChIP-PCR on immunoprecipitated genomic DNA from astroglia collected 1 day or 6 days after plating.

(F) Timeline of sample collection for Western Blot analysis.

(F'-F'') Western blot analysis of total cell lysate from early and prolonged cultured astroglia. Equal amounts of protein were loaded onto gels and blotted for REST and LaminB (loading control). Note that REST protein levels do not increase during astrocyte maturation in these culture conditions. (F'') Histogram depicting the quantification of the Western blot results as shown in (F') from three independent biological samples with ImageJ, two-tailed unpaired t-test n.s= $p>0.05$.

(G) Schematic representation of the conditional REST mutant allele. The second exon (black square) is flanked by loxP sites (red triangles). The green triangle is the remaining FRT site after neo cassette removal.

(H) Experimental design for early or late REST deletion by Cre in an adenoviral vector added to the cultured astrocytes as indicated.

(H') Western blot of total cell lysate from early and delayed cultures. Cells were transduced with an adenoviral vector serving as a control and lacking Cre (lane 1and 3) or Cre-encoding adenovirus (lane 2 and 4) for 48 hours. Equal amounts of protein were loaded onto gels and blotted for REST and LaminB (loading control for nuclear proteins). Lower band (empty arrowhead)

is a truncated form of REST lacking exon2, unable to bind DNA (see Figure S6I).

(I) Histogram depicting REST μChIP-PCR on immunoprecipitated genomic DNA from REST-cKO astrocytes collected 48 hours after infection with Cre-expressing adenovirus. No significant difference was observed between mock ChIP and REST-ChIP. The experiments were performed in parallel to those in Fig. 6B: note that in Fig. 6B the enrichment is 4-5 time higher, indicating an almost complete loss of REST expression.

(J) Timeline of mRNA analysis following Cre-mediated REST deletion.

(J') Histogram depicting the expression of selected genes determined by real-time qPCR upon early or delayed REST deletion. Note that *NeuroD1* and *NeuroD4* are expressed at higher levels upon early REST deletion but not following late REST ablation.

*Figure S7. Related to Figure 7.*

*GFAP expression upon REST deletion.*

(A-D) Examples of GFAP (white) expression in $REST^{flox}$ astrocytes transduced with Neurog2ERT2 (red), Cre (green) in the absence (A) or presence of (OHT) according to the scheme in Figure 7 (A,B early deletion; C,D delayed deletion). Scale bar: 100$\mu$m.

(E) Histogram depicting the proportion of Ascl1-regulated genes common to a specific time point analysed in the present study and genes regulated in (Wapinski et al., 2013), compared to genes regulated at a specific time point.

(F) Venn diagram showing genes regulated by Ascl1ERT2 in our analysis at any time and Ascl1 in Wapinski et al., 2013 (see Table S7 for gene list).

(G) GO term analysis (Biological Processes) performed on the common genes at any time (212).

**Supplemental Table legends**

**Table S1, related to Figure 1**. Overview of regulated genes upon Neurog2ERT2 induction.

**Table S2, related to Figure 1**. Gene Ontology (GO) terms associated with genes regulated by Neurog2ERT2 after 4 hours of OHT treatment. Analysis was performed using DAVID analysis tool. Cut off pValue<0.05.

**Table S3, related to Figure 1**. Overview of regulated genes upon Ascl1ERT2 induction.

**Table S4, related to Figure 1**. GO terms associated with genes regulated by Ascl1ERT2 after 4 hours of OHT treatment. Analysis was performed using DAVID analysis tool. Cut off pValue<0.05.

**Table S5, related to Figure 1**. GO terms associated with genes regulated by both Neurog2ERT2 and Ascl1ERT2 after 24 hours of OHT treatment. Analysis was performed using DAVID analysis tool. Cut off pValue<0.05.

**Table S6, related to Figure 1**. Overview of probesets regulated by Neurog2ERT2 and Ascl1ERT2 after 24 of OHT treatment. Pan-neuronal genes are indicated in red, genes associated with neurons with some regional differences in orange, or genes not directly associated with neuronal expression in black.

**Table S7, related to Figure 7**. Overview of genes commonly regulated by Ascl1ERT2 after OHT at any time and genes regulated by Ascl1 in Wapinski et al. 2013. Genes in red are associated with nervous system expression, in blue genes associated with neuronal expression, and in black genes not directly related to neuronal expression.

**Table S5**

Overview of GO terms associated with genes regulated by both Neurog2ERT2 and Ascl1ERT2 after 24 hours of OHT treatment, related to Figure 1

| **Category** | **GO number** | **Term** | **PValue** | **Fold Enrichment** | **Genes** |
|---|---|---|---|---|---|
| SP_PIR_KEYWORDS | | neurogenesis | 4,29E-04 | 13,77623457 | SEMA5A, HES5, SOX11, NEUROD4, SLIT1 |
| SP_PIR_KEYWORDS | | differentiation | 8,71E-04 | 6,037584541 | SEMA5A, HES5, SOX11, NEUROD4, HES6, CBFA2T3, SLIT1 |
| GOTERM_MF_FAT | GO:0005509 | GO:0005509~calcium ion binding | 0,004706859 | 3,615782313 | SMOC2, NPTX1, CADM3, TESC, LRP8, SYT7, SLIT1, CALM1 |
| SP_PIR_KEYWORDS | | calcium | 0,008647342 | 3,799300806 | SMOC2, NPTX1, CADM3, TESC, LRP8, SYT7, CALM1 |
| SP_PIR_KEYWORDS | | developmental protein | 0,014192435 | 3,411902812 | SEMA5A, HES5, SOX11, NEUROD4, HES6, PROX1, SLIT1 |
| SP_PIR_KEYWORDS | | methylation | 0,017070488 | 7,181096028 | GNG4, RASD1, RASD2, CALM1 |
| GOTERM_BP_FAT | GO:0030182 | GO:0030182~neuron differentiation | 0,020559345 | 4,602045655 | SEMA5A, HES5, NEUROD4, SLIT1, TUBB3 |
| GOTERM_CC_FAT | GO:0030054 | GO:0030054~cell junction | 0,022229501 | 4,434042553 | CADM3, LIMA1, 9030425E11RIK, SYT7, HOMER2 |
| GOTERM_MF_FAT | GO:0030528 | GO:0030528~transcription regulator activity | 0,030442265 | 2,518455342 | MYCL1, HES5, RCOR2, SOX11, NEUROD4, HES6, CBFA2T3, PROX1 |
| GOTERM_MF_FAT | GO:0003700 | GO:0003700~transcription factor activity | 0,045757161 | 2,935493373 | MYCL1, RCOR2, SOX11, HES6, CBFA2T3, PROX1 |
| SP_PIR_KEYWORDS | | prenylation | 0,049635458 | 8,208735632 | GNG4, RASD1, RASD2 |

## Supplementary Experimental Procedures

*Cell Culture*

Astrocytes were expanded in uncoated plastic flasks, and, after one week harvested using trypsin/EDTA (Gibco) and plated onto poly-D-lysine (Sigma-Aldrich) coated glass coverslips in 24-well plates (BD Biosciences) at a density of 50,000 cells per well or in uncoated 3 cm dishes (Nunc) at a density of 100,000 cells per dish in fresh and complete astrocyte medium (DMEM/F12, FBS 10%, B27, EGF 10ng/ml and FGF2 10ng/ml, penicillin/streptomycin). Depending on the experiment, cells were either infected with retroviral particles 4-12 hours after plating or transfected with plasmids 24 hours after seeding; one day later, medium was replaced with differentiation medium (DMEM/F12 medium enriched with GlutaMAX (Gibco), penicillin/streptomycin and B27 supplement (Gibco)) to allow neuronal differentiation.

Mouse embryo fibroblasts were grown in complete medium (DMEM, FBS 10%, penicillin/streptomycin). After 3 to 4 passages, cells were harvested using trypsin/EDTA (Gibco) and plated onto poly-D-lysine (Sigma-Aldrich) coated glass coverslips in 24-well plates (BD Biosciences) at a density of 25,000 cells per well in complete medium, and, after 4-12 hours, transduced with retroviruses. 24-36 hours later, complete medium was removed and differentiation medium was added. To improve survival, cells were treated with Forskolin (Sigma) and Dorsomorphin (Sigma) as described (Liu et al., 2013).

Human astrocytes were expanded in Astromedium (ScienCell) and plated onto poly-L-lysine (2$\mu$g/ml) at a density of 50,000 cells/well in 24-well plates BD Biosciences). Cells were transduced with retroviruses 4-12 hours after plating and, one day later, the medium was replaced with differentiation medium. At 8 DPI, cells were fixed and analyzed by immunostaining. Cortical neurons were isolated from E14.5 embryos as previously described (Heins et al., 2002), and 40,000 cells were plated onto reprogrammed cultures.

*Cloning procedures*

Atoh8 cDNA, kindly provided by Prof. R. Kageyama, was excised from pCLIG-Atoh8 (Inoue et al., 2001), cloned into pENTRY1A (Life Technology) and, then, recombined with the retroviral plasmid pCAG-(Dest)-ires-GFP; Prox1 cDNA, a gift of Dr. P.K. Politis (Kaltezioti et al., 2010), was excised from pEGFP-Prox1, cloned into pENTRY4 and subsequently recombined with the retroviral plasmid pCAG-(Dest)-ires-GFP ; Insm1 cDNA, from Prof. W. Huttner (Farkas et al., 2008) was excised from pTOPO-Insm1, cloned into pENTRY4 and subsequently recombined with the retroviral plasmid pCAG-(Dest)-ires-GFP; Hes6, purchased from OpenBiosystems (ThermoScientific), was recombined from pCMVSPORT-Hes6 into pDONR221 (Life Technologies) after removing the 3'UTR, and subsequently recombined with the retroviral plasmid pCAG-(Dest)-ires-GFP; NeuroD4 coding sequence, purchased from OpenBiosystems (ThermoScientific), was excised from pCMVSPORT-NeuroD4, cloned into pENTR1A and recombined with the retroviral plasmid pCAG-(Dest)-ires-Dsred; Sox4 and Sox11 are gift from Prof. D. Chichung Lie (Mu et al., 2012), Trnp1 (Stahl et al., 2013).

*Micro-Chromatin immunoprecipitation (μChIP) and qPCR*

Around 100,000 cells per sample were fixed sequentially with di(N-succimidyl) glutarate and 1% formaldehyde in phosphate-buffered saline, then lysed in 100μl of SDS lysis buffer (0.5% SDS, 10mM EDTA, 50mM Tris-HCl pH8.0) supplemented with protease inhibitor cocktail (Roche), sonicated for 10 min, and diluted 5 times in IP buffer (0.2M HEPES pH8.0, 2M NaCl, 0.02M EDTA, 0.1% Na-DOC, 1% Triton X-100, 1mg/ml BSA, protease inhibitor cocktail). Immunoprecipitations (for antibodies and dilutions see Supplementary Experimental procedures) were followed by 5X washing with modified RIPA buffer (50mM HEPES pH7.6, 1mM EDTA, 1% NP-40, 1% Na-DOC, 0.5M LiCl) 5X and with Tris-EDTA buffer (10mM Tris-HCl pH8, 1mM EDTA) 1X and elution with 50 μl of IP elution buffer (50mM NaHCO3, 1% SDS) twice with vortexing for 15 min which were then combined to a clean tube to reverse cross-linking with 0.45M NaCl at 95°C for 15 min. Samples were treated with

proteinase K and purified using QIAquick MINelute column (Qiagen). Quantities of immunoprecipitated DNA were calculated by comparison with a standard curve generated by serial dilutions of input DNA using a 7500 Real-Time PCR System (Applied Biosystems) and a SYBR green-based kit for quantitative PCR (iQ Supermix, Bio-Rad). The data were plotted as means of at least three independent ChIP assays and two independent amplifications.

Antibodies and dilutions used for micro-Chromatin immunoprecipitation: rabbit anti-H3K4me3 (0.25 μg per ChIP sample; Millipore, 07-473), mouse anti-H3K27me3 (0.25 μg per ChIP sample; Abcam, ab6002), mouse anti-H4K20me3 (0.75 μg per ChIP sample; ActiveMotif, clone 6F8-D9), mouse anti-5'mC (0.4 μg per ChIP sample; Abcam, ab10805), rabbit anti-5'hmC (0.4 μg per ChIP sample; ActiveMotif, 39791), rabbit anti-REST (0.75 μg per ChIP sample; Millipore, 07-579) or rabbit anti-HA (0.5 μg per ChIP sample; Abcam, ab9110).

Below the list of primers used for ChIP experiments:

| Gene Name | Primer forward | Primer Reverse |
|---|---|---|
| Atoh8_enh | GAGCCAGCCAAAGTGCTAAC | GGACACAGCCAGATGGTCTT |
| Cnpy3 | CTGGGTAACCACGGCAAC | TCCTCTCATTGGCTATAAAGCAG |
| Dll1 ORF | GTCTCAGGACCTTCACAGTAG | GAGCAACCTTCTCCGTAGTAG |
| Insm1_intron | GTGTCCGCTGAGTCCTTCC | AGAACGCAGTGCCCATCTT |
| Neurod1_prom | GAACCACGTGACCTGCCTAT | GTCCGCGGAGTCTCTAACTG |
| Neurod1_REST | TAACTGATTGCACCAGCCCTTCCT | ACTCGGTGGATGGTTCGTGTTTGA |
| Neurod4_prom2 | AAAAGGAGACCAGACCAGCA | GGGTGGGGTGTAACAGATTG |
| Neurod4_enh | TACTGTGGGGGTGGGAGTAG | CTAGGGCAAGCTAGGGAAGA |
| Neurod4_REST | CCCGCGAGTAGTTCTTTCAG | CTACCCTGTGGGCAACATCT |
| Prox1_prom | CCAGGACGCAGGTCTTTTT | TACTTTTCCGAGCCTTCCTC |
| Sox11_prom | TTCAAAAGAAATCCGCGAGT | ATCTGCACTGGGGTTCAGTC |
| Trnp1_enh | CCATACCCACAATCCCTCTG | GCCAAAGGACCAGAGTTGTG |

*Transfection of mouse postnatal astroglia cultures*

For transfection DNA-liposome complexes were prepared in Optimem medium (Invitrogen) using the retroviral plasmids previously described and Lipofectamine 2000 (L2K, Invitrogen). Astrocyte cultures were exposed to DNA-liposome complexes at a concentration of 0.6 *μ*g DNA with 0,75*μ*l L2K per 400 *μ*l of OptiMEM medium for 4 hours; then the transfection medium was replaced by fresh astrocyte medium and, one day later, the medium was changed with the differentiation medium.

*Viral particle production*

Viral vectors were produced with a vesicular stomatitis virus pseudotype at titers of $10^{8-11}$ as determined on HEK cells, and cells were infected about 12 hours after splitting as described above (Heinrich et al., 2011). Cells were allowed to differentiate accordingly to the experiment performed.

*MicroRNA generation, validation and subcloning*

Gene specific MicroRNAs were designed using the Invitrogen miRNA Designer Center (https://rnaidesigner.lifetechnologies.com/rnaiexpress/setOption.do?designOption=mirna) and cloned in pCDNA6.2eGFP according to manufacturer's instructions. To test microRNA knock-down efficiency, HEK cells were transfected using Lipofectamine2000 (Invitrogen) with a vector encoding for the gene of interest and a DsRed-expressing control plasmid, and alternatively with a control plasmid (GFP), a scramble-eGFP-miRNA or gene-specific-eGFP-miRNA. 48 hours later, cells were harvested, RNA was extracted using RNeasy MicroPlus Kit (Qiagen), quantified, retro-transcribed and analyzed by real time quantitative PCR as described previously. Gene expression was evaluated on the basis of cDNA content (human Gapdh expression, ΔΔCt method (Livak and Schmittgen, 2001) and normalized to the transfection efficiency (DsRed expression). Each sample was analyzed in triplicate. Primers used are listed below.

*Immunocytochemistry*

Cells were fixed in 4 % paraformaldehyde (PFA) in phosphate buffered saline (PBS) for 10 min at room temperature (RT), washed in PBS and pretreated in 0.5% Triton X-100 in PBS for 30 min, followed by incubation in 3% BSA and 0.5% Triton X-100 in PBS for 30 min. Primary antibodies were incubated on specimens at room temperature for 2 hours in 2% BSA, 0.5% Triton X-100 in PBS. The following primary antibodies were used: anti-Neurog2 (monoclonal anti-IgG2a, kindly provided by Dr. David Anderson, Howard Hughes Medical

Institute); anti-Flag (polyclonal, rabbit 1: 400, Sigma-Aldrich, F7425); anti-Green Fluorescent Protein (polyclonal GFP, chicken, 1:400, Aves Labs, GFP-1020); anti-Glial Fibrillary Acidic Protein (polyclonal GFAP, rabbit, 1:1000, DakoCytomation, Z0334; mouse monoclonal IgG1, 1:300, Sigma-Aldrich NB G3893); anti-GLAST (ACSA1, Milteny Biotec, 1:300, 130-095-822); Anti-Aldh1l1 (monoclonal IgG1, 1:200, Millipore MAB N495); anti-Red Fluorescent Protein (RFP, polyclonal rabbit, 1:1000, Rockland 600-401-379; rat monoclonal IgG2a 1:300, Chromotek); anti βIII-tubulin (mouse IgG2b, 1:500, Sigma, T8660); anti MAP2 (polyclonal rabbit, 1:300, Miltenyi Biotec, AB 5622; mouse monoclonal IgG1, 1:300, Millipore, MAB 378); anti-vGlut1 (polyclonal rabbit, 1:300, Synaptic Systems, 135302); anti-vGAT (polyclonal guine pig, 1:500, Synaptic Systems, 131004); anti-LeX (monoclonal mouse, Santa Cruz, sc-21702), anti-pSMAD1-5 (polyclonal rabbit, 41D10, Cell Signaling), anti-nuclei (monoclonal mouse MAB1281, Chemicon). After washing in PBS, cells were incubated with appropriate species- or subclass-specific secondary antibodies conjugated to CyTM2, CyTM3, CyTM5 (1:500, Jackson ImmunoResearch), Alexa Fluor 488 (1:500, Invitrogen), FITC (fluorescein isothiocyanate, 1:500, Jackson ImmunoResearch), TRITC (tetramethyl rhodamine isothiocyanate, 1:500, Jackson ImmunoResearch) or biotin (1:500, Jackson ImmunoResearch or Vector Laboratories) for 2h in the dark at room temperature, followed by extensive washing in PBS. Following treatment with secondary antibodies conjugated to biotin, cells were subsequently incubated for 2h at room temperature with Alexa Fluor 405 streptavidin (1:500, Invitrogen), or stained with DAPI, then mounted with Aqua Poly/Mount (Polysciences, Warrington, PA).

*Microarray Analysis*

Total RNA, isolated with the RNeasy Micro Plus Kit, was analyzed with The Agilent 2100 Bioanalyzer and only high quality RNA (RIN>7) was used for microarray analysis. Total RNA (120 ng) was amplified using the one-cycle MessageAmp Premier labeling kit (Ambion). 10 $\mu$g of amplified aRNA were hybridized on Affymetrix Mouse Genome 430 2.0 arrays containing about

45,000 probe sets. Staining and scanning were done according to the Affymetrix expression protocol Expression console (v.1.2, Affymetrix) was used for quality control and to obtain annotated normalized RMA data (standard settings including quantile normalisation). Statistical analysis of the microarrays was performed by utilizing the statistical programming environment R (C., 2005) implemented in CARMAweb (Rainer et al., 2006). Genewise testing for differential expression was done employing the paired limma *t*-test. p-values<0.01 were used to define sets of regulated genes, which were further filtered for fold-changes>1.2x and average expression in one group>10. Heatmaps were generated with CARMAweb.

Below the list of primers used for the Real Time qPCR:

| Gene Name | Accession number | Primer forward | Primer reverse | Probe # | Ampl. Length |
|---|---|---|---|---|---|
| Atoh8 | NM_153778.3 | tcagcttctccgagtgtgtg | tagcctgtggcaggtcact | 29 | 91 |
| Atoh8-Cod | NM_153778.3 | caaagccctgcagcagac | ggagtagcacggcacctg | 73 | 112 |
| Cnr1 | NM_007726.3 | gggcaaatttccttgtagca | ggctcaacgtgactgagaaa | 79 | 130 |
| Dll3 | NM_007866.2 | ctgcctgatggcctcgta | gctgctctctccaggtttca | 7 | 85 |
| Dlx2 | NM_010054.2 | gcctcacccaaactcaggt | aggcacaaggaggagaagc | 1 | 126 |
| Gapdh (human) | NM_002046 | agccacatcgctcagacac | gcccaatacgaccaaatcc | 66 | 60 |
| Gapdh (mouse) | BC083065 | ttcaccaccatggagaagg | cacacccatcacaaacatgg | 29 | 102 |
| Hes6 | NM_019479.3 | acggatcaacgagagtcttca | ttctctagcttggcctgcac | 66 | 72 |
| Insm1 | NM_016889.3 | ggtttgctctgcctaccaat | tcacccaaaacaacccgta | 108 | 62 |
| Lmo1 | NM_057173 | ccggcgtgactacctgag | aagctgggatcagcttgc | 27 | 71 |
| NeuroD1 | NM_010894.2 | cgcagaaggcaaggtgtc | tttggtcatgtttccacttcc | 1 | 90 |
| NeuroD4 | NM_007501.4 | actactcgcgggagctgac | ccatccaggattgtgtgttg | 22 | 104 |
| NeuroD4-cod | NM_007501.4 | aactggggcctcaatctacc | agtcacaaattgaagatttttcctc | 80 | 60 |
| Phf6 | NM_027642.1 | ggaaattaaaagaggcacaaagc | gttttcacatcacagccaatg | 1 | 78 |
| Prox1 | NM_008937.2 | cgacatctcaccttattcagga | ttgcctttttcaagtgattgg | 4 | 68 |
| pvalb | NM_013645 | ggcaagattggggttgaag | agcagtcagcgccacttag | 83 | 63 |
| Sfrp1 | NM_013834.3 | atgtgctccagaagcagacc | gtcagagcagccaacatgc | 80 | 60 |
| Sox11 | NM_009234.6 | gagctgagcgagatgatcg | gaacaccaggtcggagaagt | 20 | 60 |
| Trnp1 | NM_001081156.2 | agtcagctgggggtccat | atgcagaagtcagtccagacc | 110 | 87 |
| Trnp1-Cod | NM_001081156.2 | ctgcaccgagtcttcttgg | gcgcacccttcttgagac | 71 | 133 |

*Microscopy and quantification*

Immunostainings were analyzed with a LSM710 laser-scanning confocal or Axio Observer Z1 epifluorescence microscope (Carl Zeiss). Digital images were captured using the ZEN2009-2011 software (Carl Zeiss). Retroviral vector-transduced cells or transfected cells were quantified from more than 30 randomly chosen 20x fields in at least 3 independent experiments. Branch quantification was performed using the plug-in "Simple Neurite Tracer" (Longair et al., 2011) for the image software in Fiji (Schindelin et al., 2012).

*Electrophysiology*

For electrophysiological recordings, coverslips with reprogrammed cells were transferred to an organ bath mounted on the stage of an upright microscope (Axioscope FS, Zeiss, Göttingen, Germany). Cells on coverslips were perfused with a bathing solution consisting of (in mM): NaCl 150, KCl 3, $CaCl_2$ 3, $MgCl_2$ 2, 4-(2-hydroxyethyl)-1-piperazineethanesulfonic acid (HEPES) 10, and D-glucose 10. The pH of the solution was adjusted to 7.4 (NaOH) and its osmolarity ranged between 309 to 313 mOsmol. The perfusion rate with bathing solution (see Main Materials and Methods) was set to 1.4 ml / min and recordings were performed at room temperature (23 – 24°C). In order to visualize the cultured cells, the microscope was equipped with differential interference contrast (DIC) optics and with epifluorescence optics for green and red fluorescence (filter sets: Zeiss BP450-490, LP520, Zeiss BP546/12, IP590). Images were taken and displayed using a software-operated CCD microscope camera (ORCA R, Hamamatsu, Herrsching, Germany).

The recorded signals were amplified (x10 or x20), filtered at 10 or 20 kHz (current clamp) and 3 kHz (voltage clamp), digitized at a sampling rate of 10 or 20 kHz and stored on a computer for off-line analysis. Data acquisition and generation of command pulses was done by means of a CED 1401 Power 3 system in conjunction with Signal6 data acquisition software (Cambridge electronic design, Cambridge, England). Data analysis was performed using IGOR Pro 6 (WaveMetrics, Lake Oswego, USA) together with the NeuroMatic IGOR plugin (www.neuromatic.thinkrandom.com). The action potential discharge pattern of the cells was investigated by injections of depolarizing current pulses (1 – 2 s), the amplitudes of which were raised in steps (5 or 10 pA) from 0 – 200 pA at a frequency of 0.1 Hz.

The electrodes for whole cell patch-clamp recordings were fabricated from borosilicate glass capillaries (OD: 1.5 mm, ID: 0.86 mm, Hugo Sachs Elektronik-Harvard Apparatus, March-Hugstetten, Germany) and filled with a solution composed of (in mM): potassium gluconate 135, KCl 4, NaCl 2, ethylene glycol-bis(2-aminoethylether)-N,N,N′,N′-tetraacetic acid (EGTA) 0.2, HEPES (potassium salt) 10, adenosine-triphosphate (magnesium salt,

ATP[Mg]) 4, sodium guanosine-triphosphate (NaGTP) 0.5, and phosphocreatine 10 (pH: 7.25 – 7.30, osmolarity: 288 – 291 mOsmol). The electrodes (resistance: 5 – 6 MW) were connected to the headstage of a npi ELC-03XS amplifier (npi, Tamm, Germany). The series resistance determined after establishment of the whole cell recording mode (9 – 17 MW) was compensated by 70 – 85%. Microscope images were corrected for contrast and brightness with Photoshop CS3 (Adobe Software Systems, Ireland).

By using the motorized microscope stage, each coverslip was scanned systematically and reprogrammed cells were identified by their simultaneous green and red fluorescence. Following membrane rupture, the cells were voltage-clamped to a holding potential of -60 mV and kept under this condition until stabilization of the holding current was achieved (3 – 5 min). Then the amplifier was switched to the current-clamp mode and the resting membrane potential was registered. Determination of the input resistance $R_N$ was performed either by measurement of the amplitude of a voltage deviation induced by a small hyperpolarizing current pulse (1 s, 5 – 10 pA) or by determining the slope of the current-voltage-curve (IV-curve). The somatic membrane time constant t was derived by fitting a dual exponential function to the voltage relaxation following cessation of a small hyperpolarizing current pulse and the total membrane capacity $C_N$ was estimated using a method described by (Zemankovics et al., 2010). The ability of the cells to generate action potentials was tested by injecting depolarizing current pulses (50 ms) with increasing current strengths (DI: 5 or 10 pA) or by depolarizing current ramps (50 ms) from 0 – 100 pA. The amplitudes of the action potentials (spikes) were measured as the difference between the resting membrane potential and the spike maximum, the spike duration was determined at half-maximum amplitude and the spike threshold was derived from a phase-plane plot (Bean, 2007). The action potential discharge pattern of the cells was investigated by injections of depolarizing current pulses (1 – 2 s), the amplitudes of which were raised in steps (5 or 10 pA) from 0 – 200 pA at a frequency of 0.1 Hz.

All chemicals and drugs were obtained from Sigma-Aldrich (Munich,

Germany) and Biotrend (Cologne, Germany), respectively. The $GABA_A$-receptor antagonist bicuculline (methiodide, 10$\mu$M), the AMPA receptor antagonist 2,3-dihydroxy-6-nitro-7-sulfamoyl-benzo[f]quinoxaline-2,3-dione (NBQX, 5-10$\mu$M), the NMDA receptor antagonist D-2-amino-5-phosphonopentanoate (D-AP5), and the sodium channel blocker tetrodotoxin (TTX) were added to the bathing solution. Data are given as mean and standard deviation (SD). Statistical comparison of 2 samples was performed by using a two-tailed unpaired t-test. Comparisons of 3 or more samples were done by means of a one-way analysis of variance (ANOVA with Bonferroni post-tests).

*Statistics*

Statistical analyses were performed with GraphPrism 4 software by using 1-way-ANOVA Bonferroni post-test or two-tailed unpaired t-test as indicated in the figures.

## References


Bean, B.P. (2007). The action potential in mammalian central neurons. Nature reviews Neuroscience *8*, 451-465.

C., T.R.D. (2005). R: A Language and environment for statistical computing.

Farkas, L.M., Haffner, C., Giger, T., Khaitovich, P., Nowick, K., Birchmeier, C., Paabo, S., and Huttner, W.B. (2008). Insulinoma-associated 1 has a panneurogenic role and promotes the generation and expansion of basal progenitors in the developing mouse neocortex. Neuron *60*, 40-55.

Heinrich, C., Gascon, S., Masserdotti, G., Lepier, A., Sanchez, R., Simon-Ebert, T., Schroeder, T., Gotz, M., and Berninger, B. (2011). Generation of subtype-specific neurons from postnatal astroglia of the mouse cerebral cortex. Nature protocols *6*, 214-228.

Heins, N., Malatesta, P., Cecconi, F., Nakafuku, M., Tucker, K.L., Hack, M.A., Chapouton, P., Barde, Y.A., and Gotz, M. (2002). Glial cells generate neurons: the role of the transcription factor Pax6. Nature neuroscience *5*, 308-315.

Inoue, C., Bae, S.K., Takatsuka, K., Inoue, T., Bessho, Y., and Kageyama, R. (2001). Math6, a bHLH gene expressed in the developing nervous system, regulates neuronal versus glial differentiation. Genes to cells : devoted to molecular & cellular mechanisms *6*, 977-986.

Kaltezioti, V., Kouroupi, G., Oikonomaki, M., Mantouvalou, E., Stergiopoulos, A., Charonis, A., Rohrer, H., Matsas, R., and Politis, P.K. (2010). Prox1 regulates the notch1-mediated inhibition of neurogenesis. PLoS biology *8*, e1000565.

Liu, M.L., Zang, T., Zou, Y., Chang, J.C., Gibson, J.R., Huber, K.M., and Zhang, C.L. (2013). Small molecules enable neurogenin 2 to efficiently convert human fibroblasts into cholinergic neurons. Nature communications *4*, 2183.

Livak, K.J., and Schmittgen, T.D. (2001). Analysis of relative gene expression data using real-time quantitative PCR and the 2(-Delta Delta C(T)) Method. Methods *25*, 402-408.

Longair, M.H., Baker, D.A., and Armstrong, J.D. (2011). Simple Neurite Tracer: open source software for reconstruction, visualization and analysis of neuronal processes. Bioinformatics *27*, 2453-2454.

Mu, L., Berti, L., Masserdotti, G., Covic, M., Michaelidis, T.M., Doberauer, K., Merz, K., Rehfeld, F., Haslinger, A., Wegner, M.*, et al.* (2012). SoxC transcription factors are required for neuronal differentiation in adult hippocampal neurogenesis. The Journal of neuroscience : the official journal of the Society for Neuroscience *32*, 3067-3080.

Rainer, J., Sanchez-Cabo, F., Stocker, G., Sturn, A., and Trajanoski, Z. (2006). CARMAweb: comprehensive R- and bioconductor-based web service for microarray data analysis. Nucleic acids research *34*, W498-503.

Schindelin, J., Arganda-Carreras, I., Frise, E., Kaynig, V., Longair, M., Pietzsch, T., Preibisch, S., Rueden, C., Saalfeld, S., Schmid, B.*, et al.* (2012). Fiji: an open-source platform for biological-image analysis. Nature methods *9*, 676-682.

Stahl, R., Walcher, T., De Juan Romero, C., Pilz, G.A., Cappello, S., Irmler, M., Sanz-Aquela, J.M., Beckers, J., Blum, R., Borrell, V.*, et al.* (2013). Trnp1 regulates expansion and folding of the mammalian cerebral cortex by control of radial glial fate. Cell *153*, 535-549.

Wapinski, O.L., Vierbuchen, T., Qu, K., Lee, Q.Y., Chanda, S., Fuentes, D.R., Giresi, P.G., Ng, Y.H., Marro, S., Neff, N.F., *et al.* (2013). Hierarchical mechanisms for direct reprogramming of fibroblasts to neurons. Cell *155*, 621-635.
Zemankovics, R., Kali, S., Paulsen, O., Freund, T.F., and Hajos, N. (2010). Differences in subthreshold resonance of hippocampal pyramidal cells and interneurons: the role of h-current and passive membrane characteristics. The Journal of physiology *588*, 2109-2132.
